# Reshaping the Tumor Microenvironment of KRAS^G12D^ Pancreatic Ductal Adenocarcinoma with Combined SOS1 and MEK Inhibition for Improved Immunotherapy Response

**DOI:** 10.1158/2767-9764.CRC-24-0172

**Published:** 2024-06-21

**Authors:** Robert J. Norgard, Pratha Budhani, Sarah A. O'Brien, Youli Xia, Jessica N. Egan, Brianna Flynn, Joshua R. Tagore, Joseph Seco, Gregory W. Peet, Ania Mikucka, Ruby Wasti, Li-Chuan Chan, Melanie Hinkel, Sandra Martinez-Morilla, Jeanine Pignatelli, Francesca Trapani, Emily Corse, Di Feng, Kaja Kostyrko, Marco H. Hofmann, Kang Liu, Abhishek S. Kashyap

**Affiliations:** 1 Cancer Immunology and Immune Modulation, Boehringer Ingelheim Pharmaceuticals, Inc., Ridgefield, Connecticut.; 2 Global Computational Biology and Digital Sciences, Boehringer Ingelheim Pharmaceuticals, Inc., Ridgefield, Connecticut.; 3 Late Stage Cancer Research, Boehringer Ingelheim RCV GmbH & Co KG, Vienna, Austria.; 4 Translational Medicine and Clinical Pharmacology, Boehringer Ingelheim Pharmaceuticals, Inc., Ridgefield, Connecticut.; 5 Translational Medicine and Clinical Pharmacology, Boehringer Ingelheim RCV GmbH & Co KG, Vienna, Austria.; 6 Cancer Pharmacology and Disease Positioning Department, Boehringer Ingelheim RCV GmbH & Co KG, Vienna, Austria.

## Abstract

KRAS inhibitors have demonstrated exciting preclinical and clinical responses, although resistance occurs rapidly. Here, we investigate the effects of KRAS-targeting therapies on the tumor microenvironment using a library of *Kras*^G12D^, p53-mutant, murine pancreatic ductal adenocarcinoma–derived cell lines (KPCY) to leverage immune-oncology combination strategies for long-term tumor efficacy. Our findings show that SOS1 and MEK inhibitors (SOS1i+MEKi) suppressed tumor growth in syngeneic models and increased intratumoral CD8^+^ T cells without durable responses. Single-cell RNA sequencing revealed an increase in inflammatory cancer-associated fibroblasts (iCAF), M2 macrophages, and a decreased dendritic cell (DC) quality that ultimately resulted in a highly immunosuppressive microenvironment driven by IL6^+^ iCAFs. Agonist CD40 treatment was effective to revert macrophage polarization and overcome the lack of mature antigen-presenting DCs after SOS1i+MEKi therapy. Treatment increased the overall survival of KPCY tumor-bearing mice. The addition of checkpoint blockade to SOS1i+MEKi combination resulted in tumor-free mice with established immune memory. Our data suggest that KRAS inhibition affects myeloid cell maturation and highlights the need for combining KRAS cancer-targeted therapy with myeloid activation to enhance and prolong antitumor effects.

**Significance:**

Combination of SOS1 and MEK inhibitors increase T cell infiltration while blunting pro-immune myeloid cell maturation and highlights the need for combining KRAS cancer-targeted therapy with myeloid activation to enhance and prolong anti-tumor effects.

## Introduction

*KRAS* mutations are one of the most prevalent oncogenic alterations in cancer, occurring in roughly 30% of all cancer types (1). In pancreatic ductal adenocarcinoma (PDAC), nearly 90% of all patients harbor a KRAS mutation (2) and these mutations are implicated in the initiation, maintenance, and progression of pancreatic tumors (3). Thus, the KRAS signaling network represents a major target for therapeutic intervention in PDAC; there is no targeted therapy approved in this indication.

Recent efforts to target KRAS have been reinvigorated by the development of KRAS^G12C^ mutant-specific inhibitors, Sotorasib and Adagrasib (4–6). Although these inhibitors have shown encouraging clinical results in non–small cell lung cancer thus far, nearly half of the patients do not experience significant tumor shrinkage and nearly all patients that initially showed a response eventually progress due to the emergence of resistance and evasion mechanisms (7, 8). Moreover, KRAS^G12C^ mutations occur in only 1% of patients with PDAC, limiting the patient population that would benefit from these targeted therapies (9, 10). KRAS^G12D^ represents 35% of KRAS mutations in PDAC (10) and a selective KRAS^G12D^ inhibitor (MRTX1133) has started phase I/II trials for non–small cell lung cancer, colorectal, and pancreatic adenocarcinoma. MRTX1133 showed promising antitumor effects in preclinical PDAC mouse tumor models, but primary refractory and posttreatment relapses were observed (11). Other indirect approaches to block KRAS signaling involve targeting downstream MAPK signaling, such as BRAF, ERK, and MEK inhibitors. But similar to newer mutant-specific inhibitors, responses are often not durable, and resistance is common (12).

The tumor microenvironment (TME) of PDAC is highly complex and composed of malignant cancer, stromal, and immune cells preclinically best covered in syngeneic mouse models or genetically engineered mouse models. The cross-talk in the TME between cancer-associated fibroblasts (CAF), myeloid cells, T cells, and tumor cells is important in development of acquired resistance and suppression of antitumor immunity (13). Single-cell RNA sequencing (scRNA-seq) has revealed the heterogeneity of CAFs and identified multiple subsets of CAF populations, with the most abundant being the myofibroblastic and inflammatory CAFs (myCAFs and iCAFs; ref. 14). Both populations have been shown to play a role in immune cell suppression and tumor progression (15–19). In addition to CAFs, KRAS mutations have been reported to regulate the TME via driving immune-suppressive myeloid cell populations, and causing dendritic cell paucity, both impairing antitumor immunity (20–22). Therefore, it is highly likely that a combination approach with KRAS and immuno-oncology (I/O) to target both tumor proliferation and antitumor immunity will be needed to circumvent KRAS-targeted resistance.

To recapitulate the heterogeneity of the TME in patients with PDAC, we utilized KPCY (Kras^LSL-G12D/+^; Trp53^LSL-R172H/+^; Pdx1-Cre; Rosa26^YFP/YFP^) tumor clones previously derived from an autochthonous mouse model of PDAC (22). The KPCY tumors are stromal rich, and clones were derived that result in both T cell “hot” and “cold” TMEs. The combination of SOS1 inhibitor (SOS1i)+MEK inhibitor (MEKi) has been previously shown to exert a strong antiproliferative effect on KRAS-driven tumors but has not yet been investigated in immune-competent mice harboring tumors with immune-suppressive TMEs (23). Using the KPCY tumor models, we show that SOS1i+MEKi are capable of effectively controlling tumor growth but tumors quickly relapse upon therapy removal. scRNA-seq revealed that SOS1i+MEKi increase M2 macrophage composition and results in decreased dendritic cell quality in the TME. Addition of CD40 agonist prolonged survival and increased efficacy in combination with SOSi+MEKi. Finally, addition of anti-PD-1/anti-CTLA-4 was needed for long-term durable responses.

Through rationale I/O combinations of anti-PD-1, anti-CTLA-4, and agonist-CD40, we were able to revert M2 macrophage polarization, increase dendritic cell maturation, and activate T cells. This combination results in complete tumor regressions with immune memory responses. Our findings provide strong rationale that the state of myeloid cells significantly influences I/O responses and highlights the need for combining KRAS cancer-targeted therapy with myeloid activating I/O to enhance and prolong antitumor effects.

## Material and Methods

### Cell Lines

Murine KPCY PDAC cell lines [2838c3 (TH2), 6694c2 (TC1), 6556c3 (TC2), 6419c5 (TC3), 6422c1 (TC4), 2699c4 (TC5)] were obtained from Dr. Stanger at University of Pennsylvania (Philadelphia, PA). Cells were cultured in DMEM (Corning #10-017-CV) supplemented with 10% heat-inactivated FBS (Genesee Scientific #25-514H), 1% GlutaMAX (Gibco #35050061), and 1% penicillin/streptomycin (Gibco #15140-122). Cell lines were routinely tested for *Mycoplasma* through Charles River Research Animal Diagnostic Services.

### Animals and Tumor Studies

Female C57BL/6J wild-type and CD45.1 mice were purchased from Jackson Laboratories. Mice were maintained in pathogen-free facilities at the Boehringer Ingelheim animal facility. All animal studies were performed in accordance with the Boehringer Ingelheim Institutional Animal Care and Use Committee. For tumor growth and survival experiments, 3.0 × 10^5^ cells in 100 µL sterile DMEM were injected into the right flank of 6 to 8 weeks old female C57BL/6 mice. For concurrent and bilateral tumor inoculation, 3.0 × 10^5^ TC3 cells and 3.0 × 10^5^ TC5 cells were inoculated on the left and right flanks, respectively. For adoptive transfer, T cells were isolated from CD45.1 female mice. T cells were isolated using the MACS Pan T cell isolation kit II (catalog no.: 130-095-130) and stained with Cell Trace Violet (C34557). Ten million cells were injected intravenously one day prior to Vehicle or SOS1i + MEKi treatment.

Mice were randomized when tumors reached 40–100 mm^3^ and tumor volume, (length × width^2^ × π)/6, was measured 3x weekly. Tumor volumes of 800 mm^3^ were used as an endpoint for survival analysis. Tumor regressions and waterfall plots were calculated benchmarking to the tumor size at randomization. Tumor growth inhibition (TGI) was calculated using the formula 100 − [(Tumor volume of treated − Initial tumor volume treated)/(mean final tumor volume control − mean initial tumor volume control) × 100].

### *In Vivo* Compounds

Clinical grade SOS1i (BI 1701963) and MEKi (BI 3011441) compounds were formulated in Gelucire Solvent [1% Tween-80(v/v) + 15% Gelucire 44/14(v/v) + 84% DI H_2_O(v/v)] and dosed at a final concentration of 50 and 0.3 mg/kg, respectively through oral gavage twice daily. For Fig. 2B–D, SOS1i (BI-3406; ref. 23) and trametinib (GSK1120212) were used by dissolving in 0.5% Natrosol + DMSO at 50 and 0.1 mg/kg, respectively. aPD-1(29F1A12) and aCTLA-4 (9D9) were dosed at 200 µg i.p. 2x weekly for three doses, and aCD40 (FGK45) was dosed at 200 µg i.p. for a single dose. IgG2b isotype control and IgG1 isotype controls were used. Antibodies were purchased from BioXCell.

### Cellular Growth Assay and Westerns

Cells were seeded at 500 cells per well in a 384-well plate with reduced serum media (2% FCS) for proliferation and seeded at 180,000 cells per well in 12-well plates for Western blots. Treatments were applied the following day using the HP D300e Digital Dispenser. After 2 hours of treatment, protein lysates were prepared using MSD Tris Lysis Buffer #R60TX-3 complemented with Thermo Fisher Scientific Halt Protease & Phosphatase Inhibitor Cocktail (#1861284). Protein separation and immunodetection by Jess (ProteinSimple by Bio-Techne). Primary antibodies were diluted as follows: pERK1/2 (#4370, Cell Signaling Technology) 1:50; ERK1/2 (#9102, Cell Signaling Technology) 1:50; pMEK1/2 (#9121, Cell Signaling Technology) 1:10; MEK1/2 (#9122, Cell Signaling Technology) 1:50. Sample loading was normalized between all samples detecting the total protein amount in each sample using the ProteinSimple kit #DM-TP01. A total of 96 hours treatment was used for proliferation measurements using the CellTiter-Glo Luminescent Cell Viability Assay (Promega). Data were analyzed and plotted with GraphPad PRISM.

### Flow Cytometry of Mouse Tumors

Tumors were harvested and processed on the gentleMACS Octo Dissociator. Digestion cocktail contained 800 µg/mL Dispase II, 100 µg/mL DNase I, and 400 µg/mL Collagenase P. Cell viability was assessed with Zombie Yellow Fixable viability dye. Surface marker staining was performed in the presence of anti-mu CD16/CD32 (BioLegend Tru Stain FcX). For intracellular staining, cells were fixed and permeabilized with eBiosicence Intracellular Fixation and Permeabilization buffer. Count Bright absolute counting beads (Invitrogen) were used for quantification. Flow cytometric analysis was performed on a BD LSR Fortessa Cell Analyzer. Antibodies used in flow analysis are described in the Key Resources Table (Supplementary Fig. S9).

#### IHC Staining and Analysis

Formalin-fixed (24 hours) paraffin-embedded tissue samples were immunostained using the BOND IHC polymer refine detection kit (DS9800). Sections were cut and transferred to the BOND RX (Leica). All subsequent steps [e.g., deparaffinization, antigen retrieval (ER2), staining, and development] were performed by the default 3,3-diaminobenzidine (DAB) IHC protocol. For CD8^+^ quantification, samples were stained with anti-CD8 antibody (CST98941). Slides were scanned and visualized using an Aperio AT2. Stained cells were counted in the total tissue size (mm^2^) and quantified with HALO 2.1 (Indica labs).

### Generation of Single-cell Sequencing Library

For single-cell sequencing, three groups of 7 mice were inoculated with TC3, TC4, and TC5, respectively. In each group, 3 mice were treated with vehicle and 4 mice were treated with SOS1i+MEKi when tumors reached 40–100 mm^3^. After 10 days of treatment, tumors were harvested, and single-cell suspensions were isolated from tumors as described in digestion above. Single-cell sequencing library preparation was performed using the 10x Genomics Chromium Controller and Chromium Next GEM Single Cell 3′ Kit v3.1 Chemistry Library, Gel Bead, and Chip Kits (10x Genomics) according to the manufacturer's protocols and instructions. A total of 10,000 cells were targeted per library and processed in parallel. Any samples with a proportion >10% of cDNA 150-400 bp, were subjected to an additional 0.6xSpri cleanup before proceeding with the remainder of library preparation. Libraries produced were checked for quality control using the Agilent Tapestation, quantified using the KAPA library Quantification Kit, diluted as appropriate, then sequenced using a NovaSeq6000 with paired-end sequencing and dual indexing as appropriate for the library preparation. Dual index libraries were sequenced using cycling conditions 28, 10, 10, and 90 for Read 1, i7 index, i5 index, and Read 2, respectively. Commercial kits are listed in the Key Resources Table (Supplementary Fig. S9).

### scRNA-seq and Data Processing

Fastq files were used as an input to CellRanger (version 7.0.0) to generate count matrices, with mm10 as the mouse genome reference and default parameters. Downstream analysis was done using Scanpy (version 1.9.3, python version 3.8.2). Cells were filtered on the basis of the following criteria: 500 < number of unique molecular identifier (UMI) counts < 150,000, 200 < number of genes expressed < 10,000, percentage of mitochondria genes < 0.2. Then doublet removal was done using scrublet (version 0.2.3) with expected_doublet_rate = number of cells/1,000*0.008 for each sample. Cell count was normalized using scanpy.pp.normalize_total with a scaling factor of 10,000 and then log1p transformed using scanpy.pp.log1p. Highly variable genes were determined using scanpy.pp.highly_variable_genes with n_top_genes = 4,000. Principle component analysis was done using scanpy.tl.pca with n_comps = 50. Neighborhood graph was calculated using scanpy.pp.neighbors with n_neighbors = 15. Uniform Manifold Approximation and Projection (UMAP) topology was determined using scanpy.tl.umap with default parameters. Cell clustering was done using scanpy.tl.leiden with resolution = 1. Major cell types were manually identified on the basis of common marker genes: Epithelial cell (*Krt7, Krt8, Krt18, Krt19, Epcam*), Endothelial cell (*Plvap, Cd34, Vwf*), Fibroblast (*Dcn, Col1a1, Col3a1*), Pericyte (*Rgs5, Acta2*), Myeloid cell (*Cd14, Itgam, Cd68*), T and natural killer (NK) cell (*Cd3d, Cd3e, Cd3g, Nkg7*), B cell (*Cd79a, Ly6d, Ms4a1*), Neutrophil (*S100a8, S100a9, G0s2*), Mast cell (*Tpsab1, Kit, Cpa3*). Subclustering analysis for fibroblast, myeloid, and T/NK cells were done subsequently to identify fine-grained subpopulations for these three major cell types. Specifically, highly variable genes (*n* = 4,000) were reselected within each cell type. Principle components (n_comps = 15, 50, 50 for fibroblast, myeloid and T/NK cell, respectively) and neighborhood graphs (n_neighbors = 15) were recalculated. UMAP projections and Leiden clustering (resolution = 0.05, 0.4, 1.2 for fibroblast, myeloid, and T/NK cell, respectively) were done within each cell type. Then subpopulation cell types were manually identified using published marker genes (Fig. 4C). Of note, sample 167 (TC5 treated with vehicle) had significantly lower tumor epithelial cell proportion (<50% out of all cells), thus was removed for all downstream analysis, resulting in 153,678 total cells, 10,610 fibroblast, 22,818 myeloid cells and 7,956 T/NK cells.

### Differential Gene Expression and Pathway Analysis

Differential gene expression analysis was done using scanpy.tl.rank_genes_groups (method = wilcoxon and *P* value adjusted by Benjamini–Hochberg) and pathway overrepresentation analysis was done using enrichr API (gene_sets = MSigDB_Hallmark_2020) accessed through gseapy (version 1.0.5). For identifying pathways enriched in tumor epithelial cells from different cell clones, vehicle-treated epithelial cells were extracted. Cells from each clone were compared with the rest of the two clones and differentially expressed genes (DEG) were determined by adjusted *P* value < 0.05 and |log fold change|>1. For identifying shared DEG in response to SOSi+MEKi treatment within tumor cells, all epithelial cells were extracted and DEG for each clone were first determined by comparing TC3 SOSi+MEKi versus TC3 Vehicle, TC4 SOSi+MEKi versus TC4 Vehicle and TC5 SOSi+MEKi versus TC5 Vehicle with adjusted *P* value < 0.05 and |log fold change|>0.8. Shared upregulated and downregulated genes were identified and subject to pathway analysis.

### Cell-Cell Communication

To evaluate the potential for cell–cell interactions between cell types of interest, we used CellChat (version 1.6.1, R version 4.1.2) to infer cell-cell communications with a curated mouse ligand-receptor database. We ran two sets of cell-cell communication analyses: one for all major cell types to understand cross-talk between tumor and non-tumor cells; the other for subpopulations of CAF, myeloid, T/NK cells only to understand cross-talks among TME. For each analysis, CellChat was run in each cell clone and treatment condition, namely TC3 Vehicle, TC3 SOSi+MEKi, TC4 Vehicle, TC4 SOSi+MEKi, TC5 Vehicle, TC5 SOSi+MEKi separately with population.size = TRUE to normalize against cell proportion in each cell group during the probability calculation and other default parameters. Results were then merged into one cellchat object for comparisons for each analysis.

### Data Availability

scRNA-seq data generated in this study are publicly available in Gene Expression Omnibus at GSE264527.

## Results

### SOS1i and MEKi Combination Treatment Suppresses KPCY Tumor Growth *In Vitro*

**FIGURE 1:**
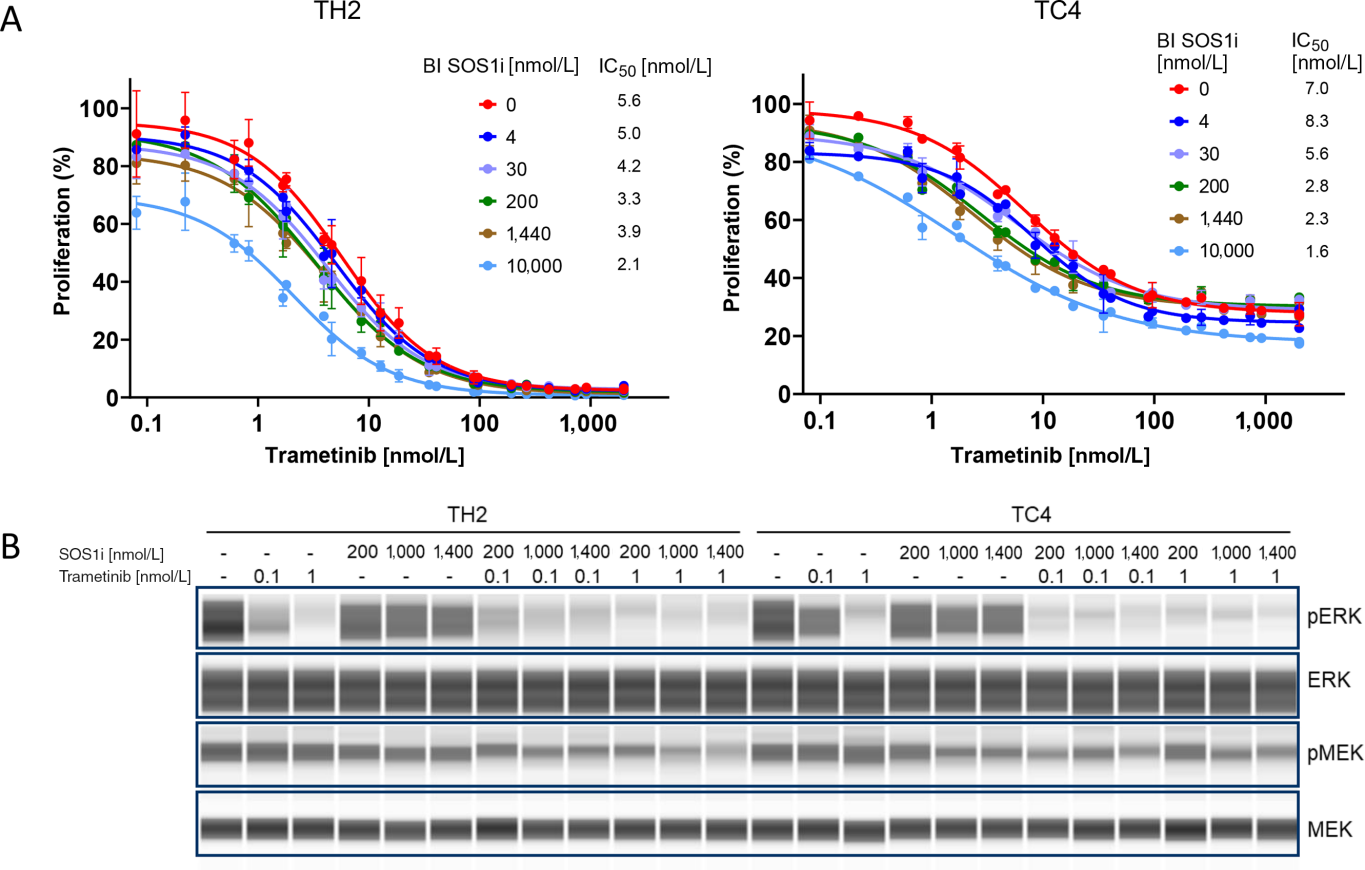
SOS1i+MEKi combination decreases proliferation and MAPK pathway activation *in vitro*. **A,** Proliferation dose–response curves in KPCY TH2 (left) and TC4 (right) cells treated with increasing concentrations of trametinib (0.08, 0.22, 0.61, 1.68, 4.62, 12.7, 35, 96.2, 264.5, 727.3, 2,000 nmol/L) and either 0.2% DMSO (red curve) or SOS1i at 4, 30, 200, 1,440 or 10,000 nmol/L. **B,** Immunoblotting for pERK, ERK, pMEK, and MEK in TH2 (left) and TC4 (right) cells treated with trametinib (0.1 or 1 nmol/L), SOS1i (200, 1,000, or 1,400 nmol/L) or the combination of the two drugs for 2 hours. SD is shown.

To understand the *in vitro* efficacy of a MEKi plus a SOS1i combination on KPCY tumor cells, we performed proliferation assays in two-dimensional monolayer conditions using two cell lines derived from subcutaneous growing tumors as published previously (22); TH2 has been shown to promote high T cell infiltration (hot) into the TME *in vivo*, and TC4 has been characterized by low T cell infiltration (cold) *in vivo* (22). The cell lines were treated with 0.8 to 1,000 nmol/L of MEKi (trametinib or tram), 0.004 to 10 µmol/L of SOS1i (BI 1701963), or the combination of the two inhibitors. Antiproliferative effects and cell viability was assessed after 96 hours. MEKi alone potently inhibited the growth of TH2 and TC4 cells with an IC_50_ of 5.6 nmol/L and 7 nmol/L, respectively (Fig. 1A). In comparison with MEKi as a single agent, addition of increasing doses of SOS1i further decreased cell viability in both cell lines and reduced IC_50_ values by 1.25- and 4-fold.

The impact of the drug treatments on MEK and ERK phosphorylation was also evaluated. TH2 and TC4 cells were treated with SOS1i (200, 1,000, or 1,400 nmol/L), MEKi (0.1 or 1 nmol/L) or the combination of both drugs for 2 hours. High pERK activity for both cell lines was observed at baseline (Fig. 1B). MEKi treatment alone resulted in a concentration-dependent pERK reduction in both cell lines. SOS1i treatment alone reduced pERK levels by approximately 50% in TH2 cells, and by 25%–40% in TC4 cells (Fig. 1B; Supplementary Fig. S1). Combined SOS1i and MEKi treatment resulted in further reduction of pERK, compared with MEKi or SOS1i treatments alone. This synergistic effect of the combination was particularly evident when SOS1i was combined with 0.1 nmol/L of MEKi, which resulted in near complete loss of pERK as compared with 40%–56% loss seen with 0.1 nmol/L MEKi alone. As expected, neither concentration of MEKi alone resulted in decreased MEK phosphorylation, likely due to the rebound of MEK activity caused by decreased ERK phosphorylation. However, in TH2 and, to a lesser extent, in TC4 cells, the addition of SOS1i resulted a concentration-dependent decrease in pMEK (Fig. 1B), suggesting that SOS1 inhibition prevents the upregulation pMEK by inhibiting the reactivation of an ERK-mediated negative feedback loop.

### SOS1i and MEKi Combination Treatment Suppresses Tumor Growth *In Vivo* and Alters T cell Infiltration in the TME of *Kras*-mutant PDAC

**FIGURE 2:**
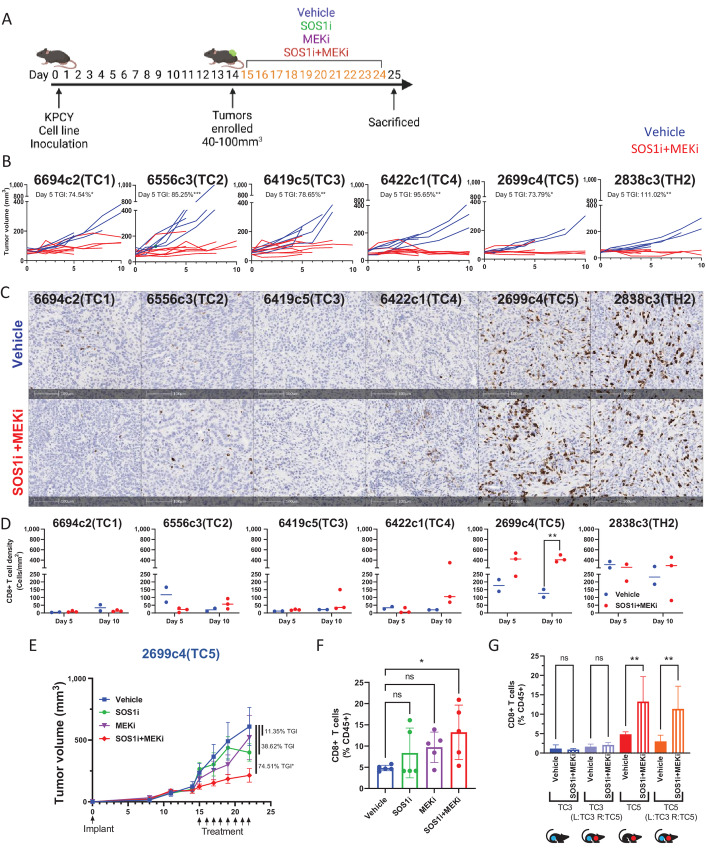
SOS1i+MEKi combination treatment suppresses KPCY tumor growth *in vivo* and alters T cell infiltration in the TME. **A,** Experiment design of treatment schedule. **B,** Tumor volume (mm^3^) of individual tumors in mice for indicated tumor clone. TGI is indicated comparing vehicle with SOS1i+MEKi for each tumor clone at day 5. Unpaired Student *t* test performed with SD shown, *n* = 3–5 per group (*<0.05, **<0.01, ***<0.001). **C,** Representative IHC images of formalin-fixed paraffin-embedded section of each KPCY lines harvested at day 10 showing CD8^+^ staining. **D,** Scatter dot plots/graph showing density of IHC CD8^+^ in the KPCY TME (as in C) of individual mice harvested at day 5 and day 10 after treatment of vehicle (blue *n* = 2) and SOS1i+MEKi (red, *n* = 3). Student *t* test performed (**<0.01). **E,** Growth curves showing tumor volume of TC5 *in vivo* after start of treatment of vehicle, SOS1i, MEKi, or combination of SOS1i+MEKi. Arrows indicate days of treatment. TGI is indicated comparing vehicle with all other treatments. Multiple comparison ANOVA performed with SD shown, *n* = 5 per group (*<0.05). **F,** Bar graph showing composition of CD8^+^ T cells in CD45^+^ cells in TC5 clone after 8 days of treatment with vehicle, SOS1i, MEKi, or combination of SOS1i+MEKi (*n* = 5). Multiple comparison ANOVA performed (* = 0.05). **G,** Bar plot summarizing CD8^+^ T cell composition as %CD45^+^ in indicated tumor. L = left flank, R = right flank. Multiple comparison ANOVA performed with SD shown (ns = nonsignificant, ** = 0.01).

To evaluate the effects of SOS1i+MEKi therapy *in vivo*, we treated C57B/L6 mice bearing established subcutaneous KPCY tumors with either a combination of SOS1i+MEKi or vehicle for 5 or 10 days (Fig. 2A). KPCY TC1–5 (T cell cold) and KPCY TH2 (T cell hot) tumors were monitored for tumor growth. At the end of the study (∼12 hours after last dose), histology was assessed for treatment-related changes. All six KPCY clones exhibited dramatic reduction in tumor growth during treatment with SOS1i+MEKi combination with a statistically significant TGI ranging from approximately 73%–110% after 5 days of twice/day dosing (Fig. 2B). Similar TGI was seen in a sustained treatment model for 18 days where the control tumors reach an average of 870 mm^3^ while SOS1i+MEKi–treated tumors were only 340 mm^3^. However, upon discontinuing SOS1i+MEKi treatment, tumors quickly relapsed and matched control tumor volumes within 5 days suggesting SOS1i+MEKi treatment does not result in sustained control of tumor cell proliferation (Supplementary Fig. S2A).

Next, we assessed the CD8^+^ T cell density in the TME using IHC to understand how SOS1i+MEKi affects the immune compartment. In KPCY TC4 and TC5, increased CD8^+^ T cell infiltration was observed after 10 days of treatment (Fig. 2C and D). This was further validated by flow cytometry (Supplementary Fig. S2B and S2C). TC1, TC2, TC3, and TH2 did not show an increase in CD8^+^ T cells. We then assessed monotherapy versus combination of SOS1i+MEKi to understand what was driving the TGI and T cell infiltration in KPCY TC5. Comparing with vehicle control, SOS1i alone or MEKi alone led to only TGI of 38.62% and 11.35%, respectively. In contrast, the combination of SOS1i+MEKi resulted in statistically significant TGI of 74.51% (Fig. 2E). Similarly, only the SOS1i+MEKi combination, but neither of the two monotherapies, induced statistically significant increase in CD8^+^ T cells in the TME (Fig. 2F).

Finally, two additional *in vivo* studies were performed to determine the trafficking of CD8^+^ T cells in response to SOS1i+MEKi treatment. To determine whether the SOS1i+MEKi treatment-induced CD8^+^ T cell increase was systemic or local, concurrent bilateral tumor inoculations were utilized. KPCY TC5 cells, the clone showing increased intratumoral CD8^+^ T cells after therapy, were subcutaneously implanted on the right flank, and KPCY TC3, the clone with no T cell increase after therapy, was implanted on the left flank (Supplementary Fig. S2D). Mice bearing established tumors were treated either with vehicle or SOS1i+MEKi combination. Following treatment, the CD8^+^ T cell composition was assessed in both tumors by flow cytometry. SOS1i+MEKi treatment led to a statistically significant increase of CD8^+^ T cells in TC5 tumors as observed earlier. The increase of T cells in TC5 tumors was not impacted by the presence of the TC3 tumor on the opposing flank of the same mouse. Likewise, TC3 tumors were not impacted by TC5 tumor on the opposing flank and remained uninfiltrated by T cells after SOS1i+MEKi (Fig. 2G). This suggested that T cell infiltration was induced only locally following SOS1i+MEKi treatment and unique to the specific TME.

To differentiate T cell infiltration versus local proliferation in response to SOS1i+MEKi treatment, KPCY TC5 tumor-bearing mice were adoptively transferred with naïve T cells from CD45.1 mice labeled with cell trace violet (CTV) one day prior to starting therapy (Supplementary Fig. S3A). Following SOS1i+MEKi treatment, a greater accumulation of CD45.1^+^CTV^+^ undivided cells were detected in the tumors compared with vehicle-treated tumors although not statistically significant. Similar amounts of CD45.1^+^CTV^−^ divided cells were detected, suggesting that SOS1i+MEKi affected T cell infiltration, not T cell proliferation. Distribution of CD45.1^+^ dividing and nondividing cells was not affected by SOS1i+MEKi treatment in draining lymph nodes, blood, and spleens of all groups. This further suggested that SOS1i+MEKi effects are localized to the TME as shown previously (Supplementary Fig. S3B). Overall, the combined SOS1i+MEKi treatment results in TGI in KPCY tumor models and furthermore affects T cell recruitment in a subset of KPCY clones.

### scRNA-seq Reveals Tumor-intrinsic Mechanisms and Cellular and Molecular Cross-talk in the TME in Response to SOS1i and MEKi Treatment

**FIGURE 3:**
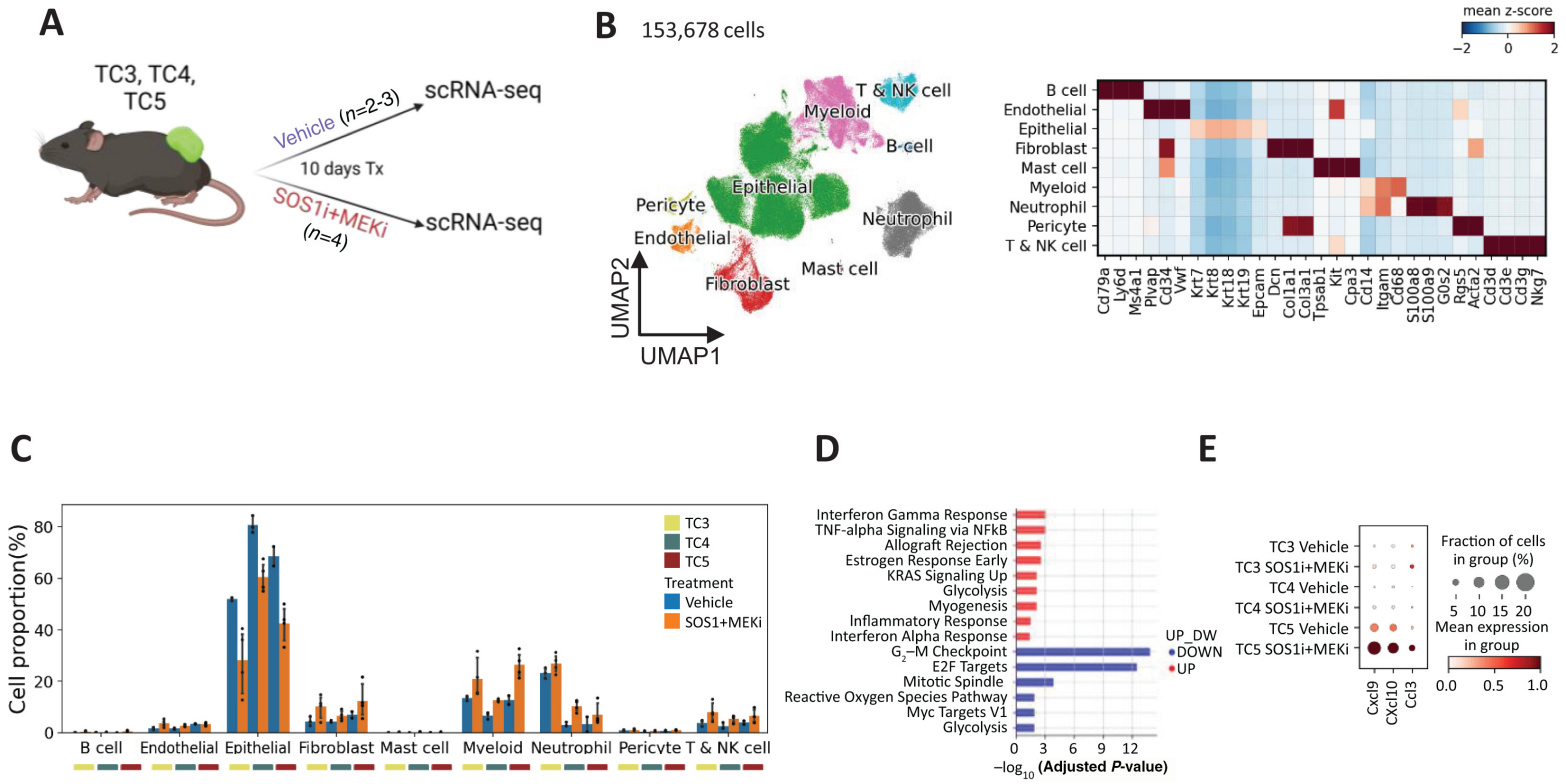
scRNA-seq reveals tumor-intrinsic mechanisms and cellular and molecular cross-talk in the TME in response to SOS1i+MEKi treatment. **A,** Experiment design of scRNA-seq experiments. **B,** UMAP visualization (left) of 153,678 KPCY TME cells from *n* = 20 mice across all conditions (TC3, TC4, and TC5 TME after treatment of vehicle or SOS1i+MEKi). Major cell types identified through graph-based clustering are indicated by color. Heat map (right) showing the relative average expression of common cell type–specific marker genes for each cluster for annotation. **C,** Bar graph showing cell proportion/composition of indicated cell type in the TME of each mouse (mean ± 95% confidence interval). **D,** Pathway enrichment analysis of upregulated and downregulated genes in epithelial/tumor cells in response to SOS1i+MEKi treatment across all mice (*n* = 20). **E,** Dot plot showing gene expression of *Cxcl9, Cxcl10*, and *Ccl3* across conditions.

To investigate the cellular and molecular mechanisms underlying SOS1i+MEKi-induced intratumoral CD8 T cell infiltration, we performed scRNA-seq on KPCY TC3, TC4, and TC5, after treatment with vehicle or the combination of SOS1i+MEKi (Fig. 3A). After quality control, we obtained 153,678 high-quality cells for downstream analysis. UMAP and clustering identified nine major cell types (Fig. 3B). Epithelial/tumor cells identified by expression of *Krt, Epcam* comprised the largest population in the TME. In addition to epithelial/tumor cells, nonmalignant TME cell groups were identified comprised of immune cells including T cells (*Cd3*), B cells (*Cd19*), myeloid cells (*Cd68*), neutrophils (*S100a8, S100a9*), mast cells (*Tpsab1, Cpa3*), stromal cells including endothelial cells (*Plvap*), and CAFs (*Dcn, Col1a1*; Fig. 3B). Tumor cells from TC3, TC4, and TC5 were transcriptionally distinctive, while nonmalignant cell types from TC3, TC4, and TC5 origin are mixed indicating their global transcriptional similarity (Supplementary Fig. S4A and S4B). Differential expressed gene and pathway analysis of vehicle-treated tumor epithelial cells revealed the intrinsic heterogeneity of tumor cells: TC3 tumor cells were enriched in KRAS signaling, IL6/JAK/STAT signaling, and IFN responses; TC4 tumor cells were enriched in epithelial mesenchymal transition and hypoxia pathways; while TC5 tumor cells were enriched in cell proliferation pathways, TNFα signaling, IL2/STAT5 signaling, and Wnt-β catenin signaling pathways (Supplementary Fig. S4C; Supplementary Tables S1–S3). Consistent with TGI *in vivo* (Fig. 2), SOS1i+MEKi reduced the proportion of tumor cells in all three KPCY lines compared with vehicle treatment (Fig. 3C).

To identify tumor-intrinsic genes and pathways that are modulated in response to SOS1i+MEKi, we first compared SOS1i+MEKi tumor cells with vehicle-treated tumor cells for TC3, TC4, and TC5 separately (Supplementary Fig. S4C; Supplementary Tables S1–S3) and then extracted the shared 165 upregulated and 37 downregulated genes across all three clones. Pathway overrepresentation analysis was performed on the basis of the shared gene lists against hallmark gene sets. The results demonstrated strong decrease in cell cycle pathways, consistent with reported function of KRAS inhibition (23, 24), while IFNγ and IFNα responses, inflammatory response, and TNFα signaling were upregulated (Fig. 3D). Interestingly, *Cd274*, or PD-L1 was upregulated after treatment in all clones (Supplementary Fig. S4D).

TC5 is classified as warm due to the presence of higher numbers of CD8^+^ T cells at baseline in tumors. Upon SOS1i+MEKi treatment, a significant amount of CD8^+^ T cells infiltrated into the TME. To understand tumor cell–specific factors that could regulate this infiltration compared with the other clones, we focused on chemokines and cytokines that significantly increased in TC5 tumors and failed to increase in nonresponsive TC3 tumors. Three factors were identified: *Cxcl9, Cxcl10*, and *Ccl3*. Upon SOS1i+MEKi, these chemokines increased further in TC5 (Fig. 3E). *Cxcl9* and *Cxcl10* are broadly associated with CD8^+^ T cell infiltration and may account for the further increase in CD8^+^ T cells in TC5 (25–28).

### SOS1i+MEKi Co-opt an Immunosuppressive Microenvironment by iCAF-M2 Macrophage Interactions and Reduced Dendritic Cell Activation

**FIGURE 4:**
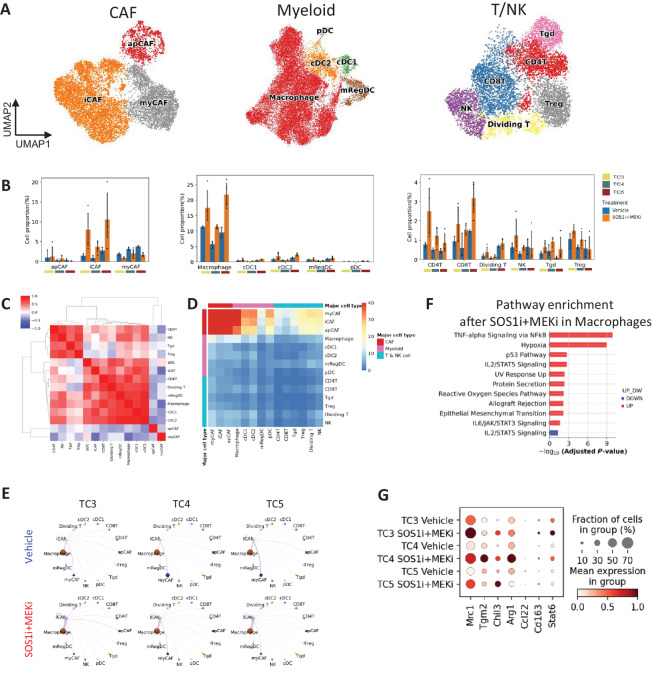
SOS1i+MEKi co-opt an immunosuppressive microenvironment by iCAF-M2 macrophage interactions and dendritic cell activation defects. **A,** UMAP visualization of subsets of CAFs, myeloid cells and T/NK cells. **B,** Bar graph showing cell proportion/composition of indicated cell subset type in the (mean ± 95% confidence interval). **C,** Heat map of frequency correlation (Pearson correlation coefficient) between every pair of nonmalignant cells in the TME. **D,** Interaction number between nonmalignant cell types. **E,** Interaction strength centered around iCAF before (top) and after SOS1i+MEKi treatment (bottom). **F,** Pathway enrichment analysis of upregulated and downregulated genes in macrophages in response to SOS1i+MEKi treatment. **G,** Dot plot showing gene expression of M2 macrophage polarization genes

To gain a better understanding of SOS1i+MEKi-induced TME modulation, we clustered and annotated subpopulations of CAF, myeloid, and T/NK cells, resulting in three CAF subpopulations: apCAF (*Cd74*), iCAF (*Col14a1, Clec3b, Has1, Il6*), and myCAF (*Lrrc15, Acta2, Tagln*); five myeloid subpopulations: macrophage (*Cd14*), cDC1 (*Clec9a*, *Xcr1*), cDC2 (*Cd209a*), mRegDC (*Ccr7, Fscn1*), and pDC (*Siglech*); six T- and NK-cell subpopulations: CD4T (*Cd4*), CD8T (*Cd8a, Cd8b1*), NK (*Ncr1*), dividing T (*Mki67*), γδ T (*Tgd*; *Trdc, Blk*), Treg (*Foxp3*; Fig. 4A; Supplementary Fig. S5A). We measured the frequencies of subpopulations per sample and the resulting bar plots revealed a differential distribution of diverse cell subsets in different KPCY TME, as well as their varying response to the SOS1i+MEKi treatment (Fig. 4B). SOS1i+MEKi increased the proportion of iCAF while decreasing the proportion of myCAF across all clones. In the myeloid cell compartment, macrophage proportions were increased across all clones while cDC1, mRegDCs, and pDCs had no change, and cDC2s were increased in KPCY TC3 and TC5 after SOS1i+MEKi.

To infer intercellular communication between cellular subsets, we performed subset composition correlation analysis that revealed the presence of two groups of cell subsets with highly correlated frequencies across the TME. Notably, iCAF, macrophage, and CD8^+^ T cells were found to be enriched in the TME following SOS1i+MEKi treatment. Interestingly, dendritic cell populations, cDC1, and cDC2 were also correlated (Fig. 4C). We then utilized CellChat (29) to further examine the expression of genes encoding ligands and receptors that can form pairs to potentiate cross-talk between cell subsets. We observed that CAF subsets harbored the highest number of ligand and receptor (L-R) pairs along with macrophages and dendritic cells (cDC1, cDC2, and pDC) also harboring L-R pairs with CAF subsets (Fig. 4D). This prompted us to further investigate how SOS1i+MEKi might influence the interactions between CAF subsets, macrophages, and dendritic cells.

To understand how SOS1i+MEKi influence CAF interactions with macrophages and dendritic cells, we calculated the L-R interaction strength that changed after treatment. Only interactions between iCAF and macrophages were notably changed after treatment (Fig. 4E; Supplementary Fig. S5B). To determine iCAF interactions with macrophages, we calculated genes and pathways that were differentially changed centered around iCAF as a ligand sender and macrophages as receptor receivers (Supplementary Fig. S5C). We found that *CCL7–CCR1/2* interactions were increased, known to play a role in myeloid cell recruitment (30, 31) and CCR2 was highly upregulated in macrophages upon SOS1i+MEKi (Supplementary Figs. S5C, S5D, and S6A).

To understand how SOS1i+MEKi treatment and the subsequent interaction with iCAFs impacts macrophages, we performed differential gene expression analysis. Pathway overrepresentation analysis on shared upregulated or downregulated genes by SOS1i+MEKi across clones revealed an increase in NFκb and IL6/STAT signaling, pathways associated with skewing macrophages toward an M2 phenotype (Fig. 4F; Supplementary Tables S4–S6). *IL6* was shown to be upregulated in iCAFs after SOS1i+MEKi (Supplementary Fig. S6B). DEGs for macrophage polarization (*Mrc1, Tgm2, Chil3, Arg1, Ccl22, Cd163, Stat6*) confirmed that after SOS1i+MEKi treatment, macrophages within the TME were skewed toward an M2 phenotype (Fig. 4G; Supplementary Fig. S6C). No change in interaction strength was found between iCAFs and dendritic cells (Fig. 4E). However, upon investigation of dendritic cell maturation markers, *CD80* and *CD40* were modulated depending on the KPCY clone. *CD86* was also downregulated in all tumor models after treatment and further suggests a blunted dendritic cell maturation (Supplementary Fig. S6D). Therefore, SOS1i+MEKi treatment results in an immunosuppressive TME with increased iCAFs, polarized M2 macrophages, and impaired dendritic cell function.

### Combination of SOS1i+MEKi Sensitizes Immunotherapy-resistant Tumors to Confer Prolonged TGI and Memory of Cold KPCY Tumors

**FIGURE 5:**
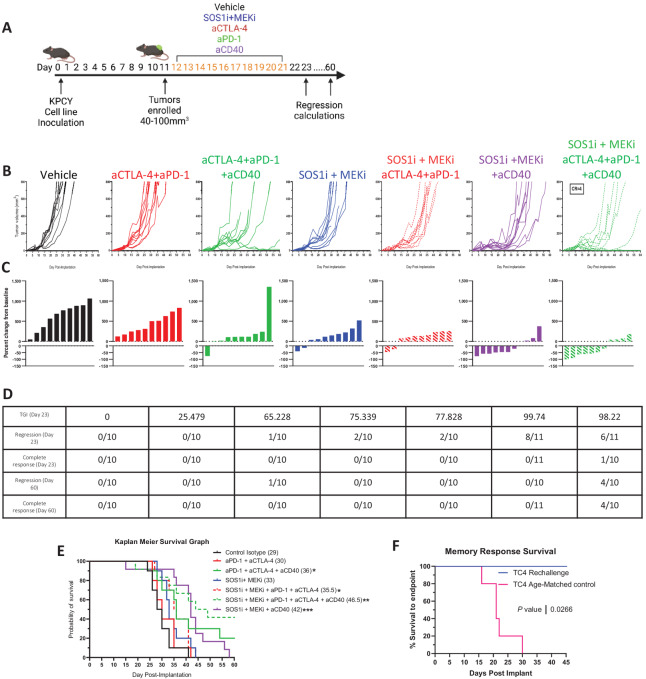
Combination of SOS1i+MEKi inhibitors sensitize cold KPCY tumors to confer prolonged TGI and immune memory. **A,** Schematic of treatment regimen of SOS1i+MEKi with aPD-1, aCTLA-4, and aCD40. **B,** Tumor volume from day postimplantation. T cell low clone (TC4) were implanted subcutaneously into C57Bl/6 mice (*n* = 10–11 mice per group) and treated when tumors reached 40–100 mm^3^. **C,** Waterfall plots from mice bearing tumors comparing start of therapy (day 12) with 48 hours after final dose (day 23; *n* = 10–11 mice per group). **D,** Table summarizing results of outcomes after treatment of TGI at day 23, tumor regressions at day 23, complete responders at day 23, tumor regressions at end of experiment day 60, and complete responders at end of experiment day 60. Columns are in order of B. **E,** Kaplan–Meier survival curves from mice implanted with TC4 clone and treated with indicated therapy. Median survival in days shown in parenthesis. *n* = 10–11 mice per group (* = 0.05, ** = 0.01, *** = 0.001). **F,** Survival of mice cured by SOS1i+MEKi+aPD-1+aCTLA-4+aCD40 therapy in B were rechallenged with a secondary tumor on the opposing flank. Kaplan–Meier survival curves of cured mice (*n* = 3) or age-matched controls (*n* = 5).

Given that SOS1i+MEKi treatment can recruit intratumoral CD8^+^ T cells into cold KPCY clones but also result in immunosuppressive skewing of myeloid compartments, we hypothesized that SOS1i+MEKi treatment may sensitize KPCY tumors to T cell rejuvenating immunotherapy with myeloid activation. To test this hypothesis, we inoculated C57BL/6 mice with TC4, a “cold” KPCY clone that has been previously shown to be unresponsive to a combination treatment including standard-of-care chemotherapy plus immunotherapy [gemcitabine, nab-paclitaxel, anti-CD40 agonist (aCD40), anti-CTLA-4 (aCTLA-4), and anti-PD-1 (aPD-1)] (22). When tumors reached a size of 40–100 mm^3^, tumors were randomized and treated for 10 days with SOS1i+MEKi plus aPD-1, aCTLA-4, and aCD40 (Fig. 5A; ref. 32). TGI, tumor regression and complete response rate were calculated after completion of all treatments for immediate responses (Study Day 23) and also 38 days after withdrawal of SOS1i+MEKi treatment (Study Day 60) for durability of response. On day 23, SOS1i+MEKi had a TGI of 75% and only 2 of 10 mice showed tumor regression (Fig. 5B–D; Supplementary Fig. S7A). Checkpoint inhibitors, aCTLA-4 and aPD-1, have minimal effect in tumor growth control. The combination of aCTLA-4-4 and aPD-1 with SOS1+MEKi was able to significantly delay tumor growth and increase survival. However, the addition of myeloid activator aCD40 with SOS1i+MEKi treatment significantly increased TGI to 99% and increased early regression rates in 8 of 11 mice. While SOS1i+MEKi+aCD40 significantly increased overall survival (Fig. 5E), there were no complete responses on study. We then added checkpoint blockade (aPD-1, aCTLA-4) to SOS1i+MEKi+aCD40. Initially, these results looked similar to the SOS1i+MEKi+aCD40 at Study Day 23, but at the end of the study 4 of 10 mice were tumor free when treated with SOS1i+MEKi+aPD-1+aCTLA-4+aCD40 (Fig. 5B–E). Of note, weight loss was observed in aPD-1+aCTLA-4+aCD40, SOS1i+MEKi+aCD40, and SOS1i+MEKi+aPD-1+aCTLA-4+aCD40, respectively. This was observed 2 days after dosing of anti-CD40, suggesting toxicity; these mice rebounded in weight over the next several days (Supplementary Fig. S7B).

To interrogate the memory T cell response, we rechallenged the remaining complete responder mice with KPCY TC4, the same tumor cell clone. Age-matched, naïve mice had rapid tumor growth. In contrast, in the complete responder mice previously treated with combination of SOS1i+MEKi+aPD-1+aCTLA-4+aCD40, 2 of 3 mice (66%) rejected the newly implanted tumor clone indicating T cell memory (Fig. 5F; Supplementary Fig. S7C).

Our data indicate that SOS1i+MEKi results in TGI, M2 myeloid cell expansion, and increased immature DCs. Targeting these myeloid populations for activation through CD40 is an essential and unique step to licensing the tumor immune response during KRAS inhibition, enhances preclinical response to SOS1i+MEKi, and can induce checkpoint blockade–mediated complete response.

## Discussion

Here, we studied the combination of SOS1 and MEK inhibitors in a library of p53-deficient, *Kras*^G12D^-driven KPCY clones with distinct transcriptional and T cell infiltration profiles. We demonstrate that combination of SOS1i+MEKi not only controls the tumor growth of murine *Kras*-p53–mutant PDAC tumors but also rewires intercellular cross-talk within the TME. The TME changes upon KRAS-targeted therapy rendering tumors vulnerable to an immunotherapy combination regimen that resulted in tumor clearance and immune memory.

It is well known that PDAC tumor cells are addicted to the KRAS oncogene, making the MAPK pathway an attractive target (24, 33). Prior research in the field has relied on MEK inhibition for treatment in KRAS-driven tumors and has assessed its role in overall antitumor immunity (34). MEKi as a single agent fails to induce a durable response (35, 36). However, multiple preclinical studies combined MEKi with other chemotherapeutic/immunotherapeutic agents, such as anti-PD-1 or anti-PD-L1, to modulate the TME, increase CD8 Teff cells, reduce MDSCs, and reduce tumor proliferation and overall tumor burden (36–39). Clinically, some BRAF^V600E^-driven melanoma and advanced cutaneous melanoma demonstrate promising outcomes with immunotherapy (40, 41), yet others (phase II MSS mCRC study) show minor partial and no complete responses (42), underlying the need to explore additional combinations to enhance the efficacy of MEK inhibition.

Previous *in vitro* and *in vivo* studies in immune-compromised mice demonstrated that SOS1i reduced the formation of GTP-loaded RAS, limited cellular proliferation of human tumors driven by various KRAS mutations, and enhanced MEK inhibition in KRAS-dependent cancers (23). Consistent with these findings, we observed sensitivity to combined SOS1i+MEKi treatment in murine KPCY cells *in vitro* and TGI *in vivo*. To understand the full impact of SOS1i+MEKi in immune-competent mice, we utilized scRNA-seq and noted downregulation of G_2_–M, E2F-regulated cell cycle, and proliferation pathways resulting in decreased tumor cell proportions *in vivo*. However, Hofmann and colleagues (23) and the current study following treatment with SOS1i+MEKi combination did not observe long-term tumor regressions in all tumor models. Therefore, we turned our attention to microenvironmental changes after SOS1i+MEKi treatment to deepen and lengthen the effects in combination with immunotherapy.

It is well appreciated that the type of TME plays a pivotal role in response to immunotherapy with the presence of CD8^+^ T cells a major indicator (43). While all KPCY clones showed *in vivo* TGI in response to SOS1i+MEKi, varying degrees of CD8^+^ T cell infiltration into these tumors were seen after treatment. This suggests differing tumor-intrinsic pathways are important for tumor growth control and local recruitment of CD8^+^ T cells, such as *CXCL9/10* upregulation in TC5. Upon withdrawal of SOS1i+MEKi, tumors immediately relapsed regardless of T cell infiltration, indicating that even in clones with infiltrated T cells, T cells were either not fully functional in killing tumor cells or a potential immunosuppressive environment had led to a dysfunctional T cell compartment. Kemp and colleagues noted a similar phenomenon using a specific KRAS inhibitor upon which T cells infiltrate but do not determine compound efficacy (11). Further evidence of immunosuppression after SOS1i+MEKi inhibition was indicated by increased PD-L1 on tumor cells and downregulation of *CD86* on dendritic cells. Stratification of patients by tumor cell expression of PD-L1 and TME type can inform and tailor combination modalities (43) and we therefore explored clones with significant CD8^+^ T cell infiltration in response to checkpoint blockade. Anti-PD-1 therapy is largely thought to be nonresponsive in patients with PDAC and other solid tumors (44). Despite a TME favorable for aPD-1 therapy with increased CD8^+^ T cells and upregulation of PD-L1 on tumor cells after SOS1i+MEKi therapy, aPD-1+aCTLA-4+SOS1i+MEKi was still ineffective in decreasing tumor growth and increasing survival.

A multitude of cell types within the microenvironment could explain the prolonged suppression T cell responses. Cellular composition changes after SOS1i+MEKi revealed an increase in fibroblasts, specifically iCAFs, and an increase in predicted cell–cell interactions between iCAFs and macrophages in all KPCY clones. Elevated levels of CCL7 and IL6 observed after SOS1i+MEKi by iCAFs is known to drive CCR2+ macrophages into tumors and induce an M2 protumorigenic macrophage phenotype (31) through IL6/JAK/STAT3 signaling (45, 46). The presence of CCR2+ M2 polarized macrophages has been shown to enable tumor recurrence (30) and clinically, inhibition of CCR2+ macrophages increases CD8^+^ T cells (47). Thus, the iCAF-induced increase in CCR2+ polarized suppressive macrophages after SOS1i+MEKi will need to be addressed for successful therapy. In addition to fibroblast and macrophage changes, SOS1i+MEKi treatment altered dendritic cells toward an immature phenotype, losing their ability to act as professional antigen-presenting cells and fully activate T cell responses (48). These *CD86* reduced dendritic cells may also explain the ineffectiveness of aPD-1 therapy as it has been shown to be required for efficacy (49).Overall, this combination of fibroblast, myeloid, and dendritic cell rewiring after treatment creates an immunosuppressive environment not conducive to prolonged T cell activation and function.

To achieve immune activation and enhance and maintain efficacy of SOS1i+MEKi treatment, we utilized our scRNA-seq insights as a roadmap for immunotherapy combinations. We applied SOS1i+MEKi alongside checkpoint immunotherapy combinations of agonistic aCD40, aPD-1, and aCTLA-4 with the rationale to: (i) reverse macrophage polarization toward an M1 phenotype, (ii) restore dendritic cell maturation and antigen presentation by utilizing agonistic aCD40, a myeloid activator (32, 50, 51), and (iii) maintain and further activate T cells inhibited by PD-L1 upregulation on tumor cells. We applied this triple immunotherapy combination to a cold tumor, normally unresponsive to this regimen (22). Only when SOS1i+MEKi was combined with aCD40, aPD-1, and aCTLA-4, complete responses with robust immune memory were observed (Supplementary Fig. S8). Interestingly, aCD40 agonist alone alongside of SOS1i+MEKi was able to improve survival but was unable to cause complete regressions. Previous studies have seen similar effects through combination of MEK inhibition and aCD40. Yet, these studies required multiple doses of aCD40 for durable responses (52). Clinical trials with agonist CD40 antibody (Selicrelumab) in patients with resectable PDAC showed similar phenotypes to our study: a shift in macrophage polarization from M2 to M1, increase in T cell proliferation and PD-1 upregulation (53). This antibody and others (APX005M Sotigalimab) have been associated with modest tumor responses with several treatment-related adverse events such as elevated liver aspartate aminotransferase/alanine aminotransferase, lymphopenia, thromboembolic events, and cytokine release syndrome (53, 54). Given the known toxicities associated with aCD40 agonism, we achieved long-term responses here with a single dose followed by three doses of T cell–targeted therapies to produce complete tumor immune responses. This result gives light to the notion that activating only T cells or only myeloid cells is not enough to drive durable antitumor immunity and suggests that agonistic aCD40 plays an indispensable and non-redundant “licensing” role in enabling anti-PD-1/anti-CTLA-4 to exert their antitumor efficacy.

Importantly, unlike KRAS mutant-specific inhibitors, SOS1 inhibition and MEK inhibition in our study is not limited to targeting KRAS pathway only in tumor cells. It is highly likely that the microenvironmental changes observed in fibroblasts, myeloid cells, and dendritic cells may result from SOS1i+MEKi direct effects in addition to the changes to the tumor cells. However, recent studies with KRAS mutant-specific inhibitors note similar TME changes suggesting that the majority of effects observed with our pan KRAS inhibitors are likely driven by on-target tumor cell-intrinsic effects (55). Kemp and colleagues showed an upregulation of fibroblasts with KRAS^G12D^ inhibition while Mahadevan and colleagues also showed an upregulation of inflammatory iCAFs after KRAS^G12D^ inhibition in late stages of tumor growth (11, 56). Interestingly, these studies, including ours, contrast with the genetic iKRAS model, in which removal of KRAS activation led to a loss of αSMA^+^ fibroblasts (24, 57, 58). However, studies utilizing specific KRAS^G12D^ inhibitors were performed in late-stage tumors, whereas the iKRAS studies were performed at early intraepithelial neoplasia stages suggesting that tumor stage may impact the phenotype.

Our finding that KRAS-driven mechanisms maintain cellular growth and immunosuppression of both adaptive and innate immunity, highlights future therapeutic strategies that should consider a combination of KRAS cancer-targeting therapy with strategies targeting both T cells and myeloid cells. However, the toxicity associated with myeloid cell activators, such as aCD40, should be considered and carefully managed or modified for TME-specific delivery. Modalities incorporating dual myeloid and T cell targeting could be considered for further therapeutic development to best impart the full potential of KRAS-targeting agents.

## Supplementary Material

Supplementary Table 1 Supplementary Table 1

Supplementary Table 2 Supplementary Table 2

Supplementary Data 3 Supplementary Table 3

Supplementary Table 4 Supplementary Table 4

Supplementary Table 5 Supplementary Table 5

Supplementary Table 6 Supplementary Table 6

Supplementary Figure 1 Supplementary Figure 1

Supplementary Figure 2 Supplementary Figure 2

Supplementary Figure 3 Supplementary Figure 3

Supplementary Figure 4 Supplementary Figure 4

Supplementary Figure 5 Supplementary Figure 5

Supplementary Figure 6 Supplementary Figure 6

Supplementary Figure 7 Supplementary Figure 7

Supplementary Figure 8 Supplementary Figure 8

Supplementary Figure 9 Supplementary Figure 9

## References

[1] Merz V , Gaule M, Zecchetto C, Cavaliere A, Casalino S, Pesoni C, . Targeting KRAS: the elephant in the room of epithelial cancers. Front Oncol 2021;11:638360. 33777798 10.3389/fonc.2021.638360 PMC7991835

[2] Singhi AD , George B, Greenbowe JR, Chung J, Suh J, Maitra A, . Real-time targeted genome profile analysis of pancreatic ductal adenocarcinomas identifies genetic alterations that might be targeted with existing drugs or used as biomarkers. Gastroenterology 2019;156:2242–53. 30836094 10.1053/j.gastro.2019.02.037

[3] Bannoura SF , MdH U, Nagasaka M, Fazili F, Al-Hallak MN, Philip PA, . Targeting KRAS in pancreatic cancer: new drugs on the horizon. Cancer Metastasis Rev 2021;40:819–35. 34499267 10.1007/s10555-021-09990-2 PMC8556325

[4] Canon J , Rex K, Saiki AY, Mohr C, Cooke K, Bagal D, . The clinical KRAS(G12C) inhibitor AMG 510 drives anti-tumour immunity. Nature 2019;575:217–23. 31666701 10.1038/s41586-019-1694-1

[5] Hallin J , Engstrom LD, Hargis L, Calinisan A, Aranda R, Briere DM, . The KRAS^G12C^ inhibitor MRTX849 provides insight toward therapeutic susceptibility of KRAS-mutant cancers in mouse models and patients. Cancer Discov 2020;10:54–71. 31658955 10.1158/2159-8290.CD-19-1167 PMC6954325

[6] Jänne PA , Riely GJ, Gadgeel SM, Heist RS, Ou S-HI, Pacheco JM, . Adagrasib in non–small-cell lung cancer harboring a KRASG12C mutation. N Engl J Med 2022;387:120–31. 35658005 10.1056/NEJMoa2204619

[7] Awad MM , Liu S, Rybkin II, Arbour KC, Dilly J, Zhu VW, . Acquired resistance to KRAS^G12C^ inhibition in cancer. N Engl J Med 2021;384:2382–93. 34161704 10.1056/NEJMoa2105281 PMC8864540

[8] Blaquier JB , Cardona AF, Recondo G. Resistance to KRAS^G12C^ inhibitors in non-small cell lung cancer. Front Oncol 2021;11:787585. 35004309 10.3389/fonc.2021.787585 PMC8739760

[9] Luo J . KRAS mutation in pancreatic cancer. Semin Oncol 2021;48:10–8. 33676749 10.1053/j.seminoncol.2021.02.003 PMC8380752

[10] Nassar AH , Adib E, Kwiatkowski DJ. Distribution of KRAS^G12C^ somatic mutations across race, sex, and cancer type. N Engl J Med 2021;384:185–7. 33497555 10.1056/NEJMc2030638

[11] Kemp SB , Cheng N, Markosyan N, Sor R, Kim I-K, Hallin J, . Efficacy of a small-molecule inhibitor of KrasG12D in immunocompetent models of pancreatic cancer. Cancer Discov 2022;13:298–311. 10.1158/2159-8290.CD-22-1066 PMC9900321 36472553

[12] Haas L , Elewaut A, Gerard CL, Umkehrer C, Leiendecker L, Pedersen M, . Acquired resistance to anti-MAPK targeted therapy confers an immune-evasive tumor microenvironment and cross-resistance to immunotherapy in melanoma. Nat Cancer 2021;2:693–708. 35121945 10.1038/s43018-021-00221-9 PMC7613740

[13] Rebelo R , Xavier CPR, Giovannetti E, Vasconcelos MH. Fibroblasts in pancreatic cancer: molecular and clinical perspectives. Trends Mol Med 2023;29:439–53. 37100646 10.1016/j.molmed.2023.03.002

[14] Elyada E , Bolisetty M, Laise P, Flynn WF, Courtois ET, Burkhart RA, . Cross-species single-cell analysis of pancreatic ductal adenocarcinoma reveals antigen-presenting cancer-associated fibroblasts. Cancer Discov 2019;9:1102–23. 31197017 10.1158/2159-8290.CD-19-0094 PMC6727976

[15] Chen Z , Zhou L, Liu L, Hou Y, Xiong M, Yang Y, . Single-cell RNA sequencing highlights the role of inflammatory cancer-associated fibroblasts in bladder urothelial carcinoma. Nat Commun 2020;11:5077. 33033240 10.1038/s41467-020-18916-5 PMC7545162

[16] McAndrews KM , Chen Y, Darpolor JK, Zheng X, Yang S, Carstens JL, . Identification of functional heterogeneity of carcinoma-associated fibroblasts with distinct IL6-mediated therapy resistance in pancreatic cancer. Cancer Discov 2022;12:1580–97. 35348629 10.1158/2159-8290.CD-20-1484 PMC9399904

[17] Krishnamurty AT , Shyer JA, Thai M, Gandham V, Buechler MB, Yang YA, . LRRC15^+^ myofibroblasts dictate the stromal setpoint to suppress tumour immunity. Nature 2022;611:148–54. 36171287 10.1038/s41586-022-05272-1 PMC9630141

[18] Mariathasan S , Turley SJ, Nickles D, Castiglioni A, Yuen K, Wang Y, . TGFβ attenuates tumour response to PD-L1 blockade by contributing to exclusion of T cells. Nature 2018;554:544–8. 29443960 10.1038/nature25501 PMC6028240

[19] Obradovic A , Graves D, Korrer M, Wang Y, Roy S, Naveed A, . Immunostimulatory cancer-associated fibroblast subpopulations can predict immunotherapy response in head and neck cancerImmunostimulatory CAF can predict immunotherapy response. Clin Cancer Res 2022;28:2094–109. 35262677 10.1158/1078-0432.CCR-21-3570 PMC9161438

[20] Lin JH , Huffman AP, Wattenberg MM, Walter DM, Carpenter EL, Feldser DM, . Type 1 conventional dendritic cells are systemically dysregulated early in pancreatic carcinogenesis. J Exp Med 2020;217:e20190673. 32453421 10.1084/jem.20190673 PMC7398166

[21] Hegde S , Krisnawan VE, Herzog BH, Zuo C, Breden MA, Knolhoff BL, . Dendritic cell paucity leads to dysfunctional immune surveillance in pancreatic cancer. Cancer Cell 2020;37:289–307. 32183949 10.1016/j.ccell.2020.02.008 PMC7181337

[22] Li J , Byrne KT, Yan F, Yamazoe T, Chen Z, Baslan T, . Tumor cell-intrinsic factors underlie heterogeneity of immune cell infiltration and response to immunotherapy. Immunity 2018;49:178–93. 29958801 10.1016/j.immuni.2018.06.006 PMC6707727

[23] Hofmann MH , Gmachl M, Ramharter J, Savarese F, Gerlach D, Marszalek JR, . BI-3406, a potent and selective SOS1–KRAS interaction inhibitor, is effective in kras-driven cancers through combined MEK inhibition. Cancer Discov 2021;11:142–57. 32816843 10.1158/2159-8290.CD-20-0142 PMC7892644

[24] Collins MA , Bednar F, Zhang Y, Brisset J-C, Galbán S, Galbán CJ, . Oncogenic Kras is required for both the initiation and maintenance of pancreatic cancer in mice. J Clin Invest 2012;122:639–53. 22232209 10.1172/JCI59227 PMC3266788

[25] Kohli K , Pillarisetty VG, Kim TS. Key chemokines direct migration of immune cells in solid tumors. Cancer Gene Ther 2022;29:10–21. 33603130 10.1038/s41417-021-00303-x PMC8761573

[26] Dangaj D , Bruand M, Grimm AJ, Ronet C, Barras D, Duttagupta PA, . Cooperation between constitutive and inducible chemokines enables T cell engraftment and immune attack in solid tumors. Cancer Cell 2019;35:885–900. 31185212 10.1016/j.ccell.2019.05.004 PMC6961655

[27] Reschke R , Yu J, Flood BA, Higgs EF, Hatogai K, Gajewski TF. Immune cell and tumor cell-derived CXCL10 is indicative of immunotherapy response in metastatic melanoma. J Immunother Cancer 2021;9:e003521. 34593622 10.1136/jitc-2021-003521 PMC8487215

[28] Neo SY , Lundqvist A. The multifaceted roles of CXCL9 within the tumor microenvironment. Adv Exp Med Biol 2020;1231:45–51. 32060845 10.1007/978-3-030-36667-4_5

[29] Jin S , Guerrero-Juarez CF, Zhang L, Chang I, Ramos R, Kuan C-H, . Inference and analysis of cell-cell communication using CellChat. Nat Commun 2021;12:1088. 33597522 10.1038/s41467-021-21246-9 PMC7889871

[30] Hou P , Kapoor A, Zhang Q, Li J, Wu C-J, Li J, . Tumor microenvironment remodeling enables bypass of oncogenic KRAS dependency in pancreatic cancer. Cancer Discov 2020;10:1058–77. 32341020 10.1158/2159-8290.CD-19-0597 PMC7334087

[31] Damme JV , Proost P, Lenaerts JP, Opdenakker G. Structural and functional identification of two human, tumor-derived monocyte chemotactic proteins (MCP-2 and MCP-3) belonging to the chemokine family. J Exp Med 1992;176:59–65. 1613466 10.1084/jem.176.1.59 PMC2119277

[32] Vonderheide RH , Glennie MJ. Agonistic CD40 antibodies and cancer therapy. Clin Cancer Res 2013;19:1035–43. 23460534 10.1158/1078-0432.CCR-12-2064 PMC3590838

[33] McCormick F . Cancer therapy based on oncogene addiction. J Surg Oncol 2011;103:464–7. 21480237 10.1002/jso.21749

[34] Blumenschein GR , Smit EF, Planchard D, Kim D-W, Cadranel J, Pas TD, . A randomized phase II study of the MEK1/MEK2 inhibitor trametinib (GSK1120212) compared with docetaxel in KRAS-mutant advanced non-small-cell lung cancer (NSCLC). Ann Oncol 2015;26:894–901. 25722381 10.1093/annonc/mdv072 PMC4855243

[35] Gupta S , Weiss A, Kumar G, Wang S, Nel A. The T-cell antigen receptor utilizes Lck, Raf-1, and MEK-1 for activating mitogen-activated protein kinase. Evidence for the existence of a second protein kinase C-dependent pathway in an Lck-negative Jurkat cell mutant. J Biol Chem 1994;269:17349–57. 7516337

[36] Datta J , Dai X, Bianchi A, Silva IDC, Mehra S, Garrido VT, . Combined MEK and STAT3 inhibition uncovers stromal plasticity by enriching for cancer-associated fibroblasts with mesenchymal stem cell-like features to overcome immunotherapy resistance in pancreatic cancer. Gastroenterology 2022;163:1593–612. 35948109 10.1053/j.gastro.2022.07.076 PMC10257389

[37] Limagne E , Nuttin L, Thibaudin M, Jacquin E, Aucagne R, Bon M, . MEK inhibition overcomes chemoimmunotherapy resistance by inducing CXCL10 in cancer cells. Cancer Cell 2022;40:136–52. 35051357 10.1016/j.ccell.2021.12.009

[38] Lee JW , Zhang Y, Eoh KJ, Sharma R, Sanmamed MF, Wu J, . The combination of MEK inhibitor with immunomodulatory antibodies targeting programmed death 1 and programmed death ligand 1 results in prolonged survival in Kras/p53-driven lung cancer. J Thorac Oncol 2019;14:1046–60. 30771521 10.1016/j.jtho.2019.02.004 PMC6542636

[39] Ebert PJR , Cheung J, Yang Y, McNamara E, Hong R, Moskalenko M, . MAP Kinase inhibition promotes T cell and anti-tumor activity in combination with PD-L1 checkpoint blockade. Immunity 2016;44:609–21. 26944201 10.1016/j.immuni.2016.01.024

[40] Hu-Lieskovan S , Mok S, Moreno BH, Tsoi J, Robert L, Goedert L, . Improved antitumor activity of immunotherapy with BRAF and MEK inhibitors in BRAFV600E melanoma. Sci Transl Med 2015;7:279ra41. 10.1126/scitranslmed.aaa4691 PMC4765379 25787767

[41] Rogala P , Czarnecka AM, Cybulska-Stopa B, Ostaszewski K, Piejko K, Ziętek M, . Long term results and prognostic biomarkers for anti-PD1 immunotherapy used after BRAFi/MEKi combination in advanced cutaneous melanoma patients. Cancers 2022;14:2123. 35565255 10.3390/cancers14092123 PMC9101360

[42] Johnson B , Haymaker CL, Parra ER, Soto LMS, Wang X, Thomas JV, . Phase II study of durvalumab (anti-PD-L1) and trametinib (MEKi) in microsatellite stable (MSS) metastatic colorectal cancer (mCRC). J Immunother Cancer 2022;10:e005332. 36007963 10.1136/jitc-2022-005332 PMC9422817

[43] Teng MWL , Ngiow SF, Ribas A, Smyth MJ. Classifying cancers based on T-cell infiltration and PD-L1. Cancer Res 2015;75:2139–45. 25977340 10.1158/0008-5472.CAN-15-0255 PMC4452411

[44] Pu N , Lou W, Yu J. PD-1 immunotherapy in pancreatic cancer: current status. J Pancreatol 2019;2:6–10.

[45] Mao X , Xu J, Wang W, Liang C, Hua J, Liu J, . Crosstalk between cancer-associated fibroblasts and immune cells in the tumor microenvironment: new findings and future perspectives. Mol Cancer 2021;20:131. 34635121 10.1186/s12943-021-01428-1 PMC8504100

[46] Xia T , Zhang M, Lei W, Yang R, Fu S, Fan Z, . Advances in the role of STAT3 in macrophage polarization. Front Immunol 2023;14:1160719. 37081874 10.3389/fimmu.2023.1160719 PMC10110879

[47] Nywening TM , Wang-Gillam A, Sanford DE, Belt BA, Panni RZ, Cusworth BM, . Targeting tumour-associated macrophages with CCR2 inhibition in combination with FOLFIRINOX in patients with borderline resectable and locally advanced pancreatic cancer: a single-centre, open-label, dose-finding, non-randomised, phase 1b trial. Lancet Oncol 2016;17:651–62. 27055731 10.1016/S1470-2045(16)00078-4 PMC5407285

[48] Deicher A , Andersson R, Tingstedt B, Lindell G, Bauden M, Ansari D. Targeting dendritic cells in pancreatic ductal adenocarcinoma. Cancer Cell Int 2018;18:85. 29946224 10.1186/s12935-018-0585-0 PMC6006559

[49] Kamphorst AO , Wieland A, Nasti T, Yang S, Zhang R, Barber DL, . Rescue of exhausted CD8 T cells by PD-1–targeted therapies is CD28-dependent. Science 2017;355:1423–7. 28280249 10.1126/science.aaf0683 PMC5595217

[50] Richman LP , Vonderheide RH. Role of crosslinking for agonistic CD40 monoclonal antibodies as immune therapy of cancer. Cancer Immunol Res 2014;2:19–26. 24416732 10.1158/2326-6066.CIR-13-0152 PMC3883444

[51] Lim CY , Chang JH, Lee WS, Kim J, Park IY. CD40 agonists alter the pancreatic cancer microenvironment by shifting the macrophage phenotype toward M1 and suppress human pancreatic cancer in organotypic slice cultures. Gut Liver 2022;16:645–59. 34933280 10.5009/gnl210311 PMC9289829

[52] Baumann D , Hägele T, Mochayedi J, Drebant J, Vent C, Blobner S, . Proimmunogenic impact of MEK inhibition synergizes with agonist anti-CD40 immunostimulatory antibodies in tumor therapy. Nat Commun 2020;11:2176. 32358491 10.1038/s41467-020-15979-2 PMC7195409

[53] Byrne KT , Betts CB, Mick R, Sivagnanam S, Bajor DL, Laheru DA, . Neoadjuvant selicrelumab, an agonist CD40 antibody, induces changes in the tumor microenvironment in patients with resectable pancreatic cancer. Clin Cancer Res 2021;27:4574–86. 34112709 10.1158/1078-0432.CCR-21-1047 PMC8667686

[54] Li D-K , Wang W. Characteristics and clinical trial results of agonistic anti-CD40 antibodies in the treatment of malignancies. Oncol Lett 2020;20:176. 32934743 10.3892/ol.2020.12037 PMC7471753

[55] Li J , Stanger BZ. The tumor as organizer model. Science 2019;363:1038–9. 30846584 10.1126/science.aau9861

[56] Mahadevan KK , McAndrews KM, LeBleu VS, Yang S, Lyu H, Li B, . KRASG12D inhibition reprograms the microenvironment of early and advanced pancreatic cancer to promote FAS-mediated killing by CD8+ T cells. Cancer Cell 2023;41:1606–20. 37625401 10.1016/j.ccell.2023.07.002 PMC10785700

[57] Collins MA , Brisset J-C, Zhang Y, Bednar F, Pierre J, Heist KA, . Metastatic pancreatic cancer is dependent on oncogenic Kras in mice. PLoS One 2012;7:e49707. 23226501 10.1371/journal.pone.0049707 PMC3513322

[58] Velez-Delgado A , Donahue KL, Brown KL, Du W, Irizarry-Negron V, Menjivar RE, . Extrinsic KRAS signaling shapes the pancreatic microenvironment through fibroblast reprogramming. Cell Mol Gastroenterol Hepatol 2022;13:1673–99. 35245687 10.1016/j.jcmgh.2022.02.016 PMC9046274

