# Supplementary material for: Reshaping the Tumor Microenvironment of KRASG12D Pancreatic Ductal Adenocarcinoma with Combined SOS1 and MEK Inhibition for Improved Immunotherapy Response
Source: Cancer Res Commun. 2024 Jun 21;4(6):1548–60. doi: 10.1158/2767-9764.CRC-24-0172 (PMC11191876; doi:10.1158/2767-9764.CRC-24-0172)
Supplement: Supplementary Figure 1 [file crc-24-0172-s07.pptx]

## Slide 1
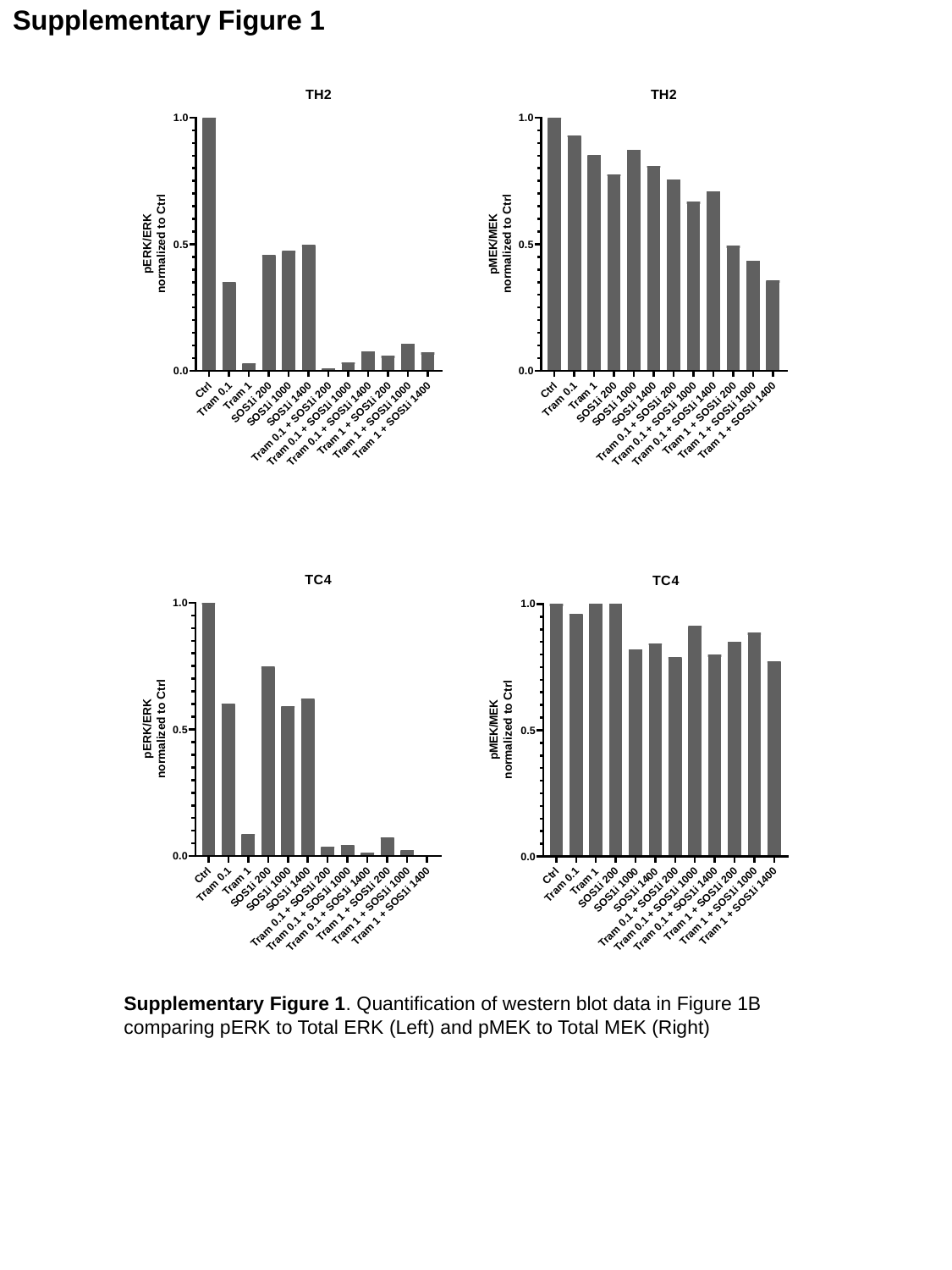

Supplementary Figure 1
Supplementary Figure 1. Quantification of western blot data in Figure 1B comparing pERK to Total ERK (Left) and pMEK to Total MEK (Right)
