# Supplementary material for: Reshaping the Tumor Microenvironment of KRASG12D Pancreatic Ductal Adenocarcinoma with Combined SOS1 and MEK Inhibition for Improved Immunotherapy Response
Source: Cancer Res Commun. 2024 Jun 21;4(6):1548–60. doi: 10.1158/2767-9764.CRC-24-0172 (PMC11191876; doi:10.1158/2767-9764.CRC-24-0172)
Supplement: Supplementary Figure 2 [file crc-24-0172-s08.pptx]

## Slide 1
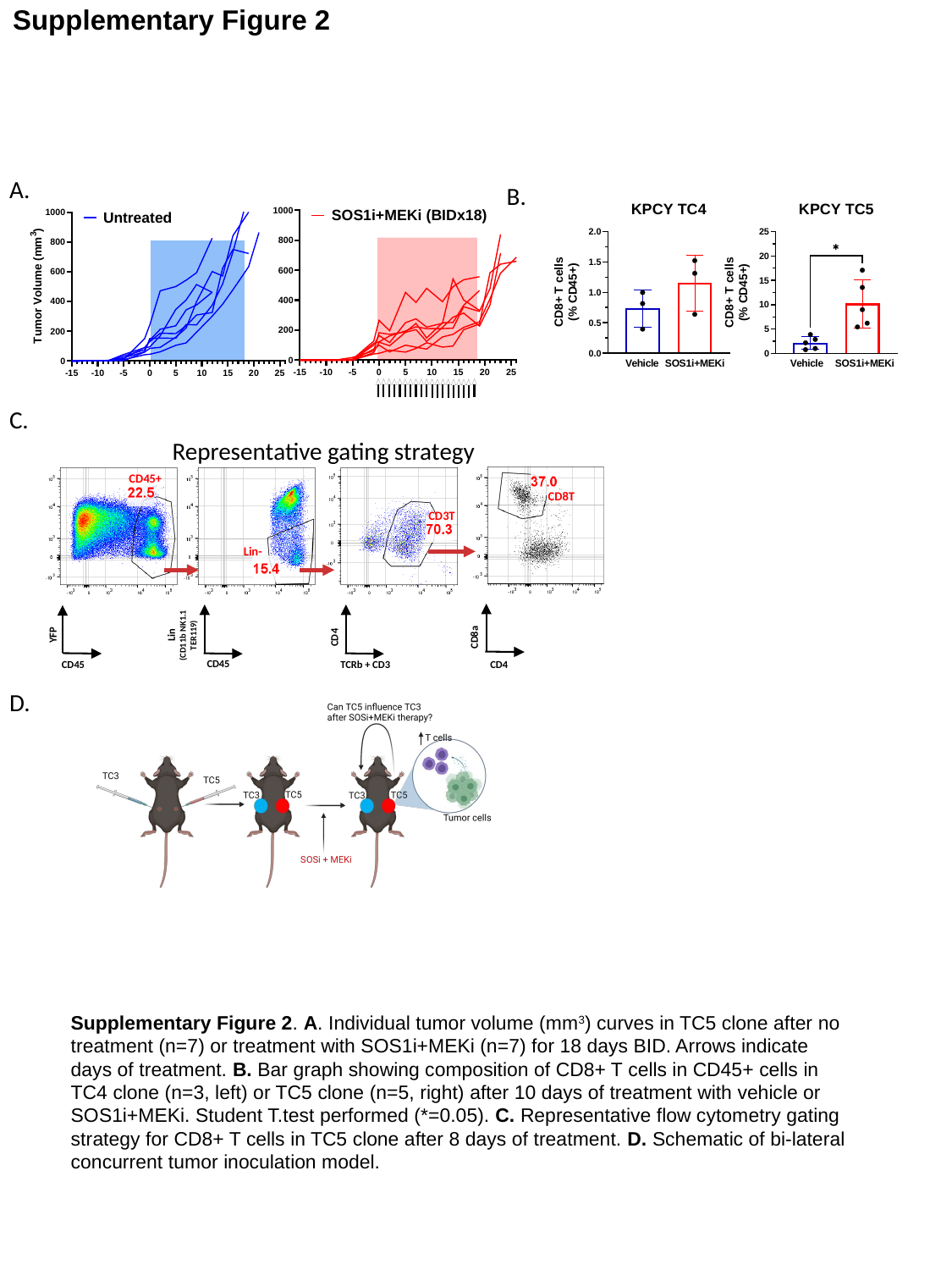

Supplementary Figure 2
A.
B.
C.
Representative gating strategy
CD45+
CD8T
CD3T
Lin-
YFP
CD8a
CD4
Lin
(CD11b NK1.1 TER119)
CD45
TCRb + CD3
 CD4
CD45
D.
Supplementary Figure 2. A. Individual tumor volume (mm3) curves in TC5 clone after no treatment (n=7) or treatment with SOS1i+MEKi (n=7) for 18 days BID. Arrows indicate days of treatment. B. Bar graph showing composition of CD8+ T cells in CD45+ cells in TC4 clone (n=3, left) or TC5 clone (n=5, right) after 10 days of treatment with vehicle or SOS1i+MEKi. Student T.test performed (*=0.05). C. Representative flow cytometry gating strategy for CD8+ T cells in TC5 clone after 8 days of treatment. D. Schematic of bi-lateral concurrent tumor inoculation model.
