# Supplementary material for: Reshaping the Tumor Microenvironment of KRASG12D Pancreatic Ductal Adenocarcinoma with Combined SOS1 and MEK Inhibition for Improved Immunotherapy Response
Source: Cancer Res Commun. 2024 Jun 21;4(6):1548–60. doi: 10.1158/2767-9764.CRC-24-0172 (PMC11191876; doi:10.1158/2767-9764.CRC-24-0172)
Supplement: Supplementary Figure 3 [file crc-24-0172-s09.pptx]

## Slide 1
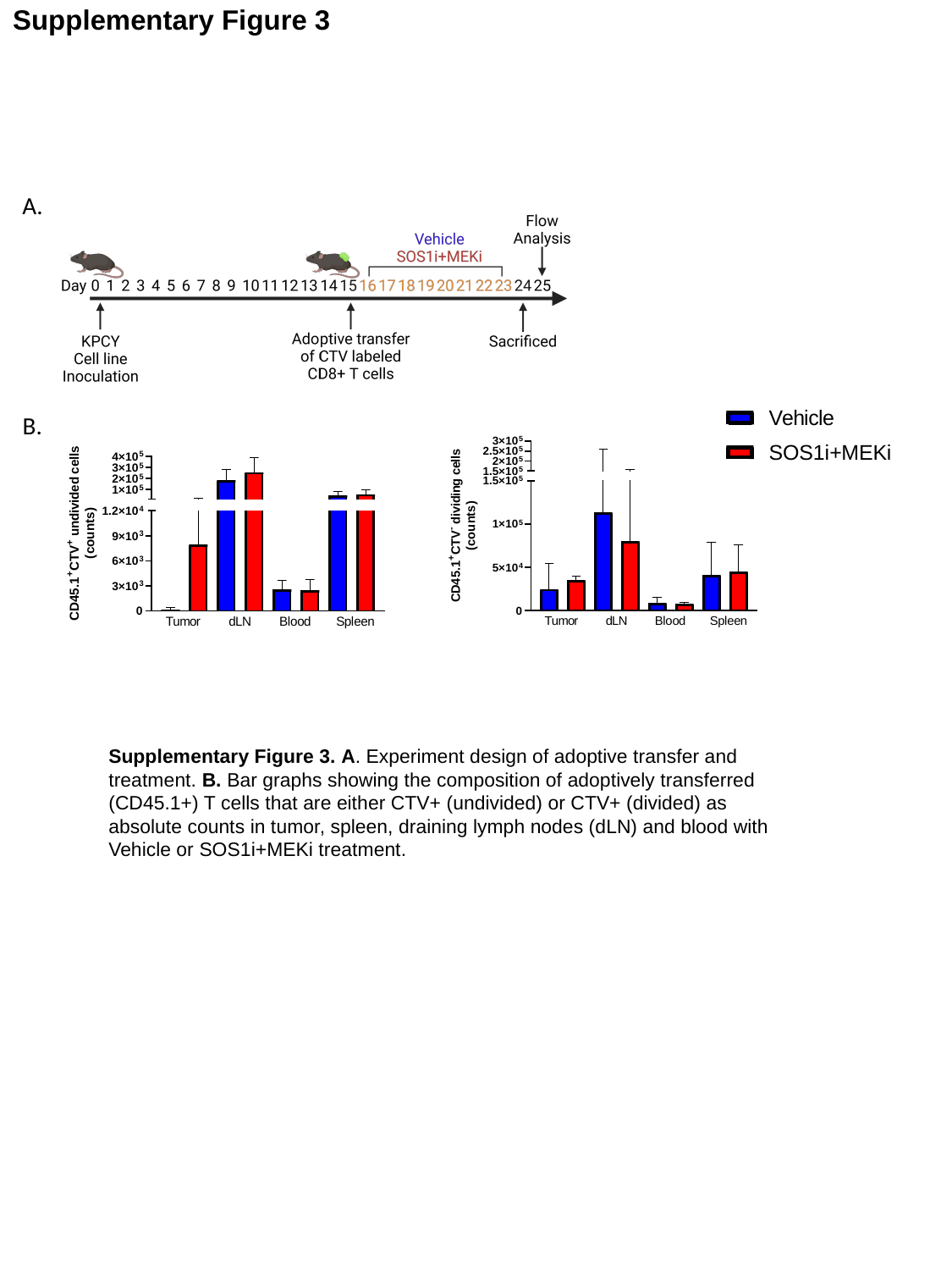

Supplementary Figure 3
A.
B.
Supplementary Figure 3. A. Experiment design of adoptive transfer and treatment. B. Bar graphs showing the composition of adoptively transferred (CD45.1+) T cells that are either CTV+ (undivided) or CTV+ (divided) as absolute counts in tumor, spleen, draining lymph nodes (dLN) and blood with Vehicle or SOS1i+MEKi treatment.
