# Supplementary material for: Reshaping the Tumor Microenvironment of KRASG12D Pancreatic Ductal Adenocarcinoma with Combined SOS1 and MEK Inhibition for Improved Immunotherapy Response
Source: Cancer Res Commun. 2024 Jun 21;4(6):1548–60. doi: 10.1158/2767-9764.CRC-24-0172 (PMC11191876; doi:10.1158/2767-9764.CRC-24-0172)
Supplement: Supplementary Figure 4 [file crc-24-0172-s10.pptx]

## Slide 1
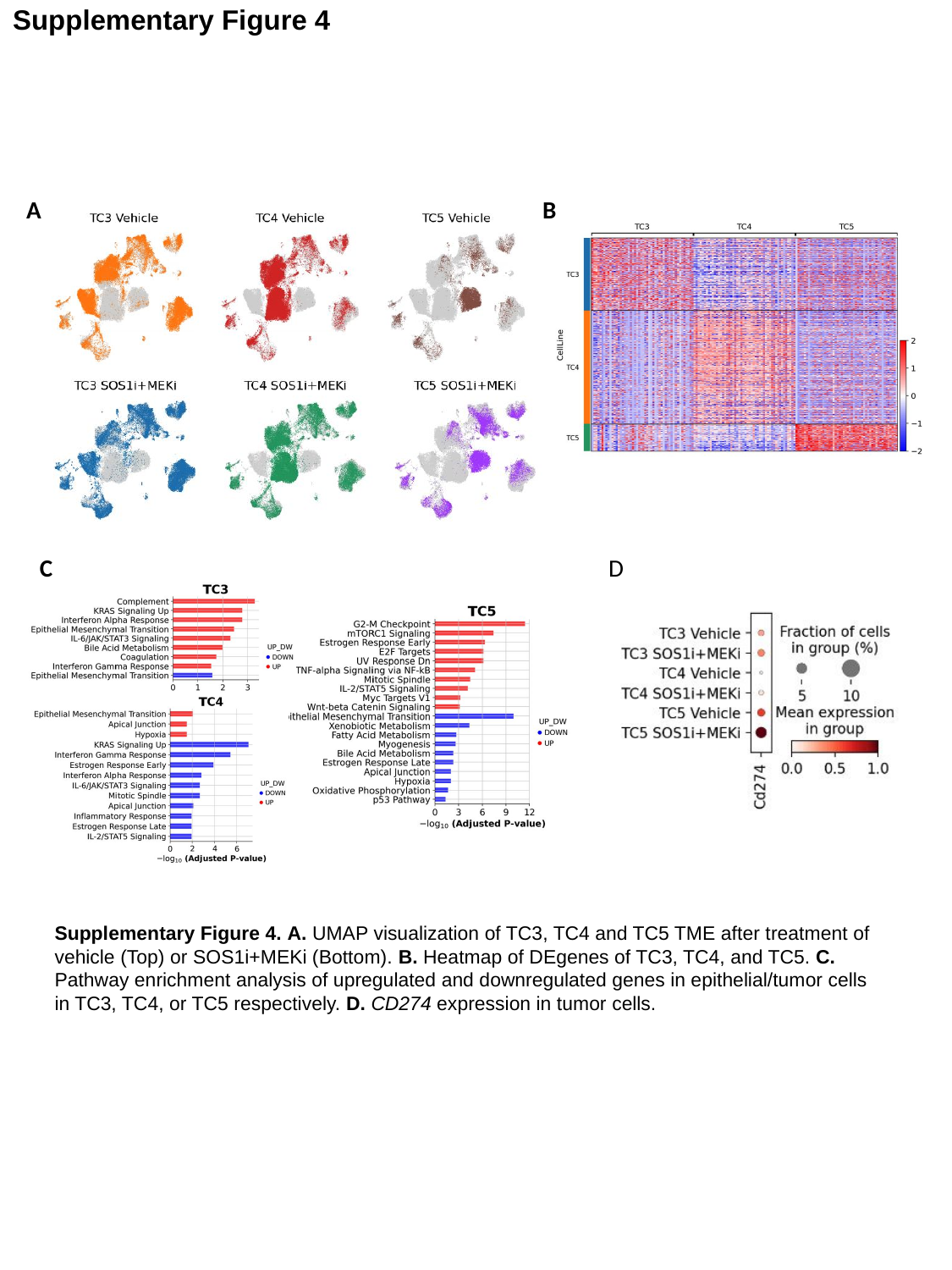

Supplementary Figure 4
A
B
C
D
Supplementary Figure 4. A. UMAP visualization of TC3, TC4 and TC5 TME after treatment of vehicle (Top) or SOS1i+MEKi (Bottom). B. Heatmap of DEgenes of TC3, TC4, and TC5. C. Pathway enrichment analysis of upregulated and downregulated genes in epithelial/tumor cells in TC3, TC4, or TC5 respectively. D. CD274 expression in tumor cells.
