# Supplementary material for: Reshaping the Tumor Microenvironment of KRASG12D Pancreatic Ductal Adenocarcinoma with Combined SOS1 and MEK Inhibition for Improved Immunotherapy Response
Source: Cancer Res Commun. 2024 Jun 21;4(6):1548–60. doi: 10.1158/2767-9764.CRC-24-0172 (PMC11191876; doi:10.1158/2767-9764.CRC-24-0172)
Supplement: Supplementary Figure 5 [file crc-24-0172-s11.pptx]

## Slide 1
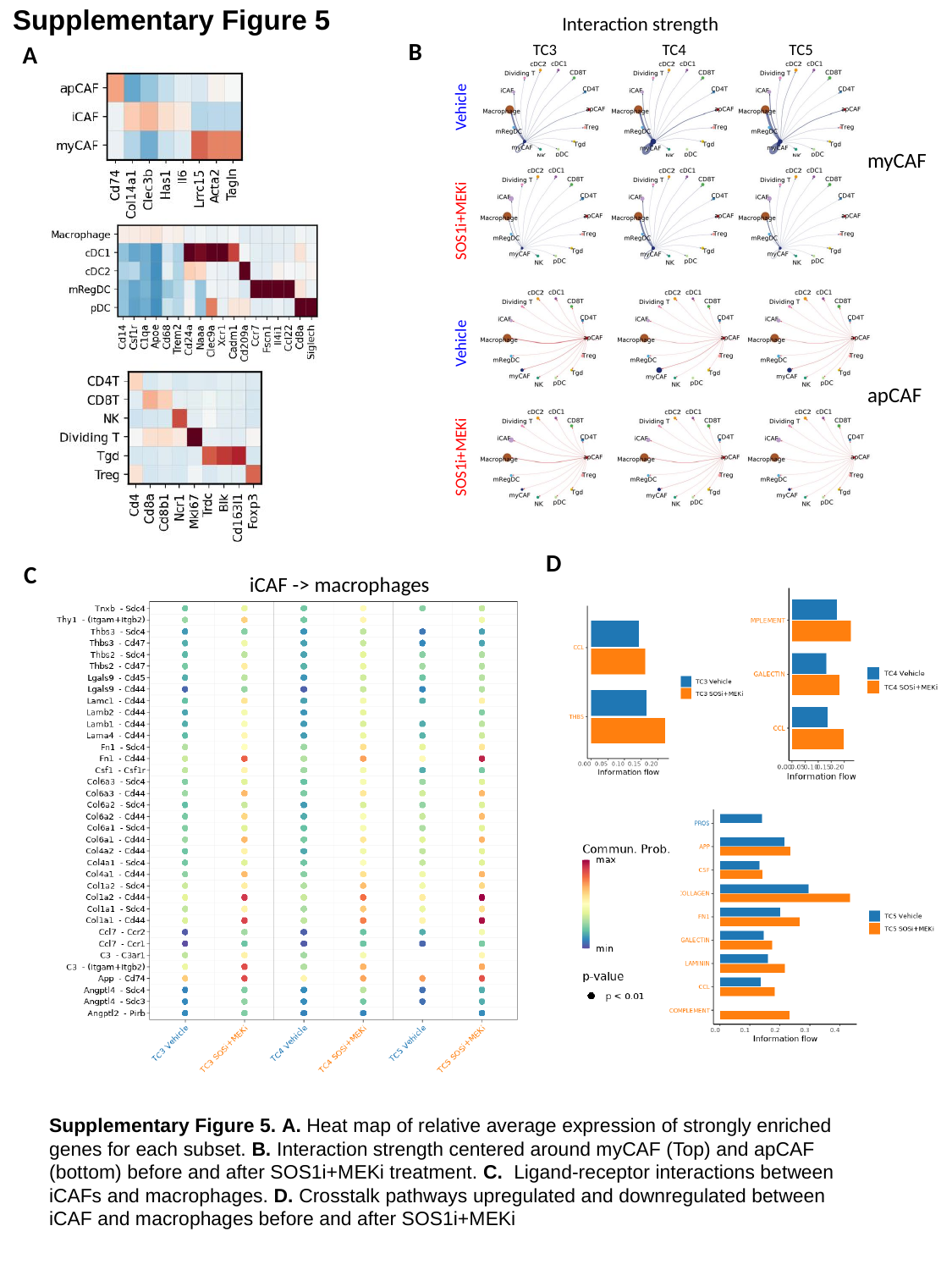

Supplementary Figure 5
Interaction strength
B
A
TC3
TC4
TC5
Vehicle
myCAF
SOS1i+MEKi
Vehicle
apCAF
SOS1i+MEKi
D
C
iCAF -> macrophages
Supplementary Figure 5. A. Heat map of relative average expression of strongly enriched genes for each subset. B. Interaction strength centered around myCAF (Top) and apCAF (bottom) before and after SOS1i+MEKi treatment. C. Ligand-receptor interactions between iCAFs and macrophages. D. Crosstalk pathways upregulated and downregulated between iCAF and macrophages before and after SOS1i+MEKi
