# Supplementary material for: Reshaping the Tumor Microenvironment of KRASG12D Pancreatic Ductal Adenocarcinoma with Combined SOS1 and MEK Inhibition for Improved Immunotherapy Response
Source: Cancer Res Commun. 2024 Jun 21;4(6):1548–60. doi: 10.1158/2767-9764.CRC-24-0172 (PMC11191876; doi:10.1158/2767-9764.CRC-24-0172)
Supplement: Supplementary Figure 6 [file crc-24-0172-s12.pptx]

## Slide 1
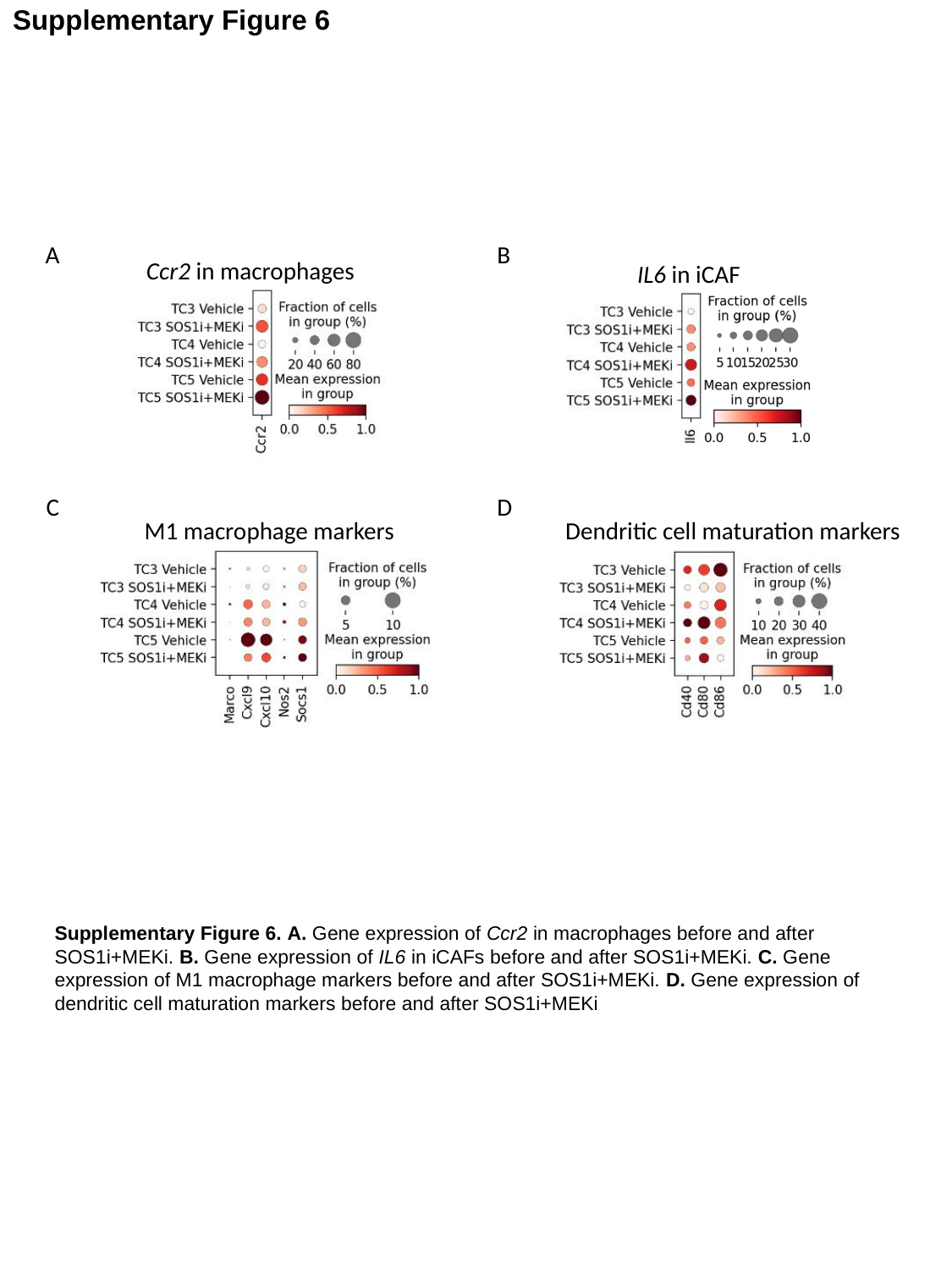

Supplementary Figure 6
A
B
Ccr2 in macrophages
IL6 in iCAF
C
D
M1 macrophage markers
Dendritic cell maturation markers
Supplementary Figure 6. A. Gene expression of Ccr2 in macrophages before and after SOS1i+MEKi. B. Gene expression of IL6 in iCAFs before and after SOS1i+MEKi. C. Gene expression of M1 macrophage markers before and after SOS1i+MEKi. D. Gene expression of dendritic cell maturation markers before and after SOS1i+MEKi
