# Supplementary material for: Reshaping the Tumor Microenvironment of KRASG12D Pancreatic Ductal Adenocarcinoma with Combined SOS1 and MEK Inhibition for Improved Immunotherapy Response
Source: Cancer Res Commun. 2024 Jun 21;4(6):1548–60. doi: 10.1158/2767-9764.CRC-24-0172 (PMC11191876; doi:10.1158/2767-9764.CRC-24-0172)
Supplement: Supplementary Figure 7 [file crc-24-0172-s13.pptx]

## Slide 1
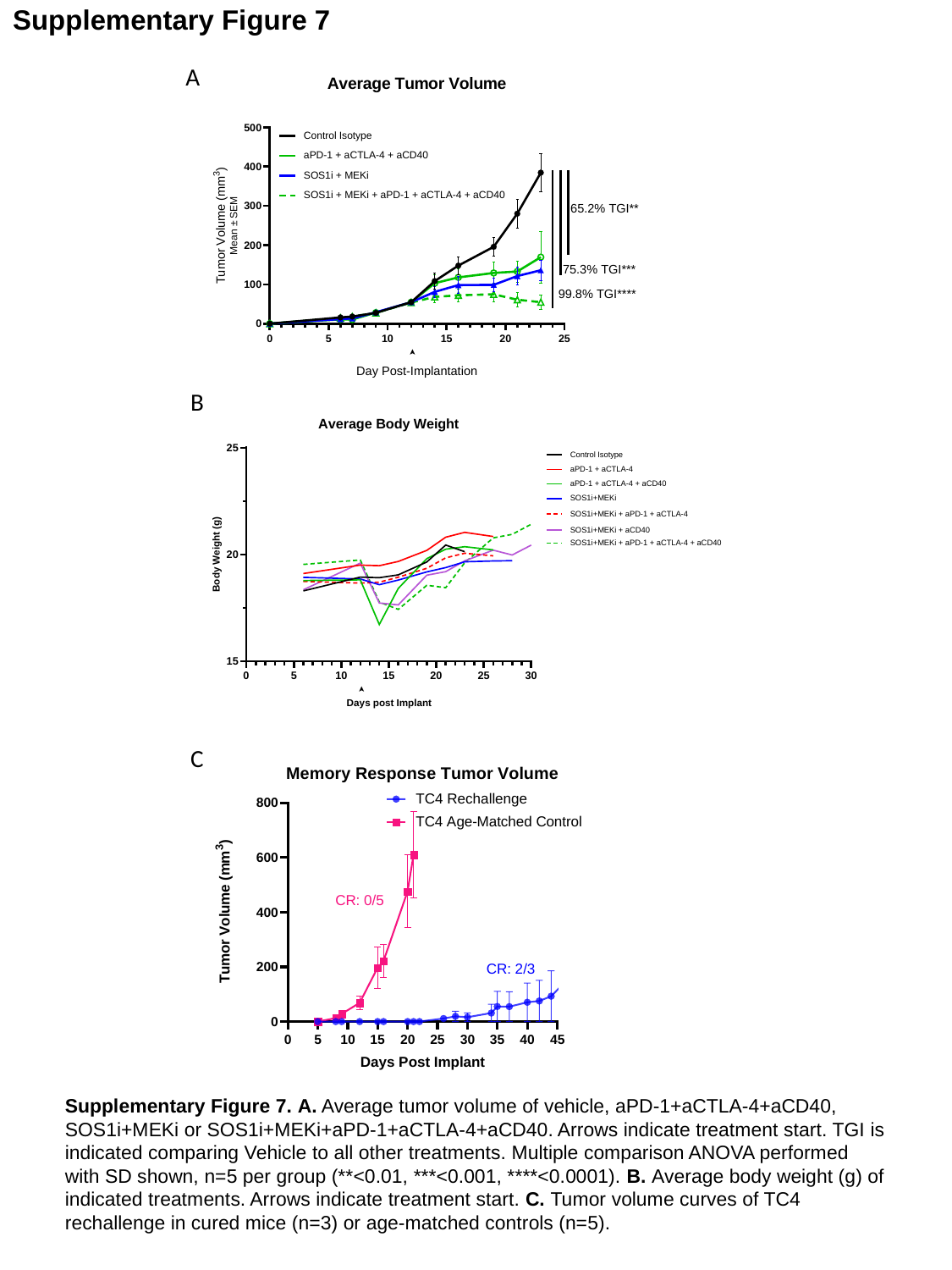

Supplementary Figure 7
A
B
C
Supplementary Figure 7. A. Average tumor volume of vehicle, aPD-1+aCTLA-4+aCD40, SOS1i+MEKi or SOS1i+MEKi+aPD-1+aCTLA-4+aCD40. Arrows indicate treatment start. TGI is indicated comparing Vehicle to all other treatments. Multiple comparison ANOVA performed with SD shown, n=5 per group (**<0.01, ***<0.001, ****<0.0001). B. Average body weight (g) of indicated treatments. Arrows indicate treatment start. C. Tumor volume curves of TC4 rechallenge in cured mice (n=3) or age-matched controls (n=5).
