# Supplementary material for: Reshaping the Tumor Microenvironment of KRASG12D Pancreatic Ductal Adenocarcinoma with Combined SOS1 and MEK Inhibition for Improved Immunotherapy Response
Source: Cancer Res Commun. 2024 Jun 21;4(6):1548–60. doi: 10.1158/2767-9764.CRC-24-0172 (PMC11191876; doi:10.1158/2767-9764.CRC-24-0172)
Supplement: Supplementary Figure 8 [file crc-24-0172-s14.pptx]

## Slide 1
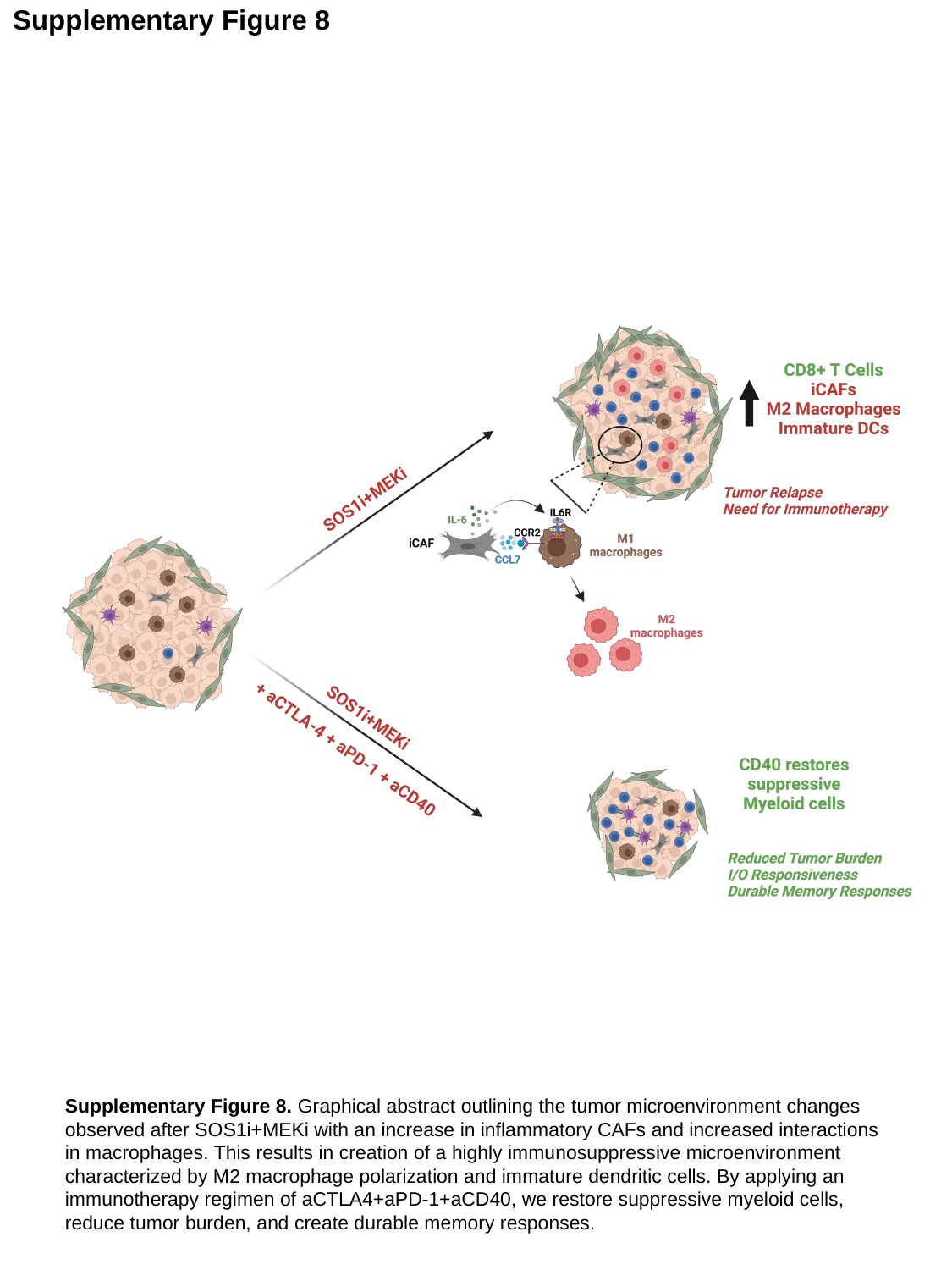

Supplementary Figure 8
Supplementary Figure 8. Graphical abstract outlining the tumor microenvironment changes observed after SOS1i+MEKi with an increase in inflammatory CAFs and increased interactions in macrophages. This results in creation of a highly immunosuppressive microenvironment characterized by M2 macrophage polarization and immature dendritic cells. By applying an immunotherapy regimen of aCTLA4+aPD-1+aCD40, we restore suppressive myeloid cells, reduce tumor burden, and create durable memory responses.
