# Supplementary material for: Reshaping the Tumor Microenvironment of KRASG12D Pancreatic Ductal Adenocarcinoma with Combined SOS1 and MEK Inhibition for Improved Immunotherapy Response
Source: Cancer Res Commun. 2024 Jun 21;4(6):1548–60. doi: 10.1158/2767-9764.CRC-24-0172 (PMC11191876; doi:10.1158/2767-9764.CRC-24-0172)
Supplement: Supplementary Figure 9 [file crc-24-0172-s15.pptx]

## Slide 1
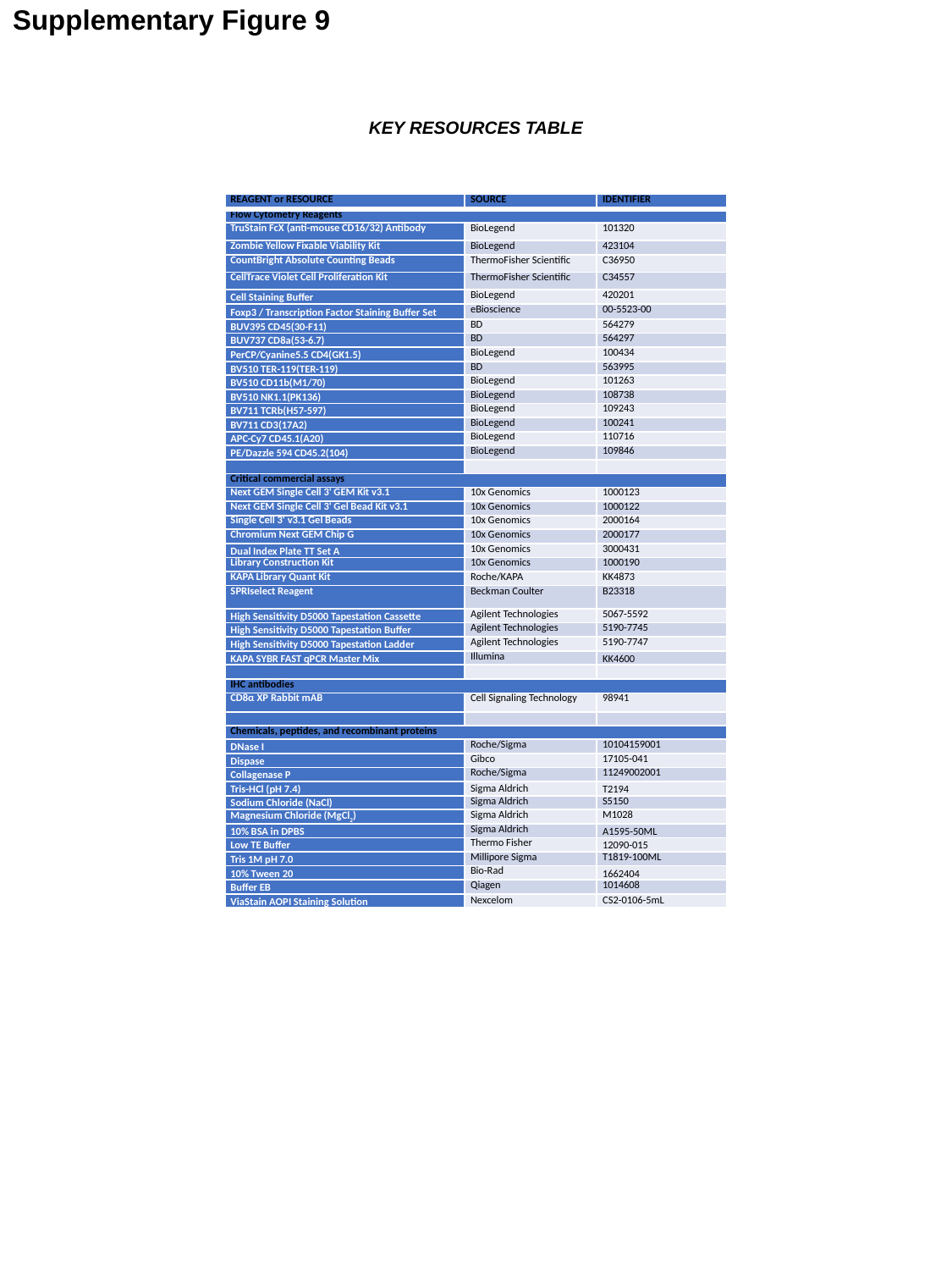

Supplementary Figure 9
KEY RESOURCES TABLE
| REAGENT or RESOURCE | SOURCE | IDENTIFIER |
| --- | --- | --- |
| Flow Cytometry Reagents | | |
| TruStain FcX (anti-mouse CD16/32) Antibody | BioLegend | 101320 |
| Zombie Yellow Fixable Viability Kit | BioLegend | 423104 |
| CountBright Absolute Counting Beads | ThermoFisher Scientific | C36950 |
| CellTrace Violet Cell Proliferation Kit | ThermoFisher Scientific | C34557 |
| Cell Staining Buffer | BioLegend | 420201 |
| Foxp3 / Transcription Factor Staining Buffer Set | eBioscience | 00-5523-00 |
| BUV395 CD45(30-F11) | BD | 564279 |
| BUV737 CD8a(53-6.7) | BD | 564297 |
| PerCP/Cyanine5.5 CD4(GK1.5) | BioLegend | 100434 |
| BV510 TER-119(TER-119) | BD | 563995 |
| BV510 CD11b(M1/70) | BioLegend | 101263 |
| BV510 NK1.1(PK136) | BioLegend | 108738 |
| BV711 TCRb(H57-597) | BioLegend | 109243 |
| BV711 CD3(17A2) | BioLegend | 100241 |
| APC-Cy7 CD45.1(A20) | BioLegend | 110716 |
| PE/Dazzle 594 CD45.2(104) | BioLegend | 109846 |
| | | |
| Critical commercial assays | | |
| Next GEM Single Cell 3' GEM Kit v3.1 | 10x Genomics | 1000123 |
| Next GEM Single Cell 3' Gel Bead Kit v3.1 | 10x Genomics | 1000122 |
| Single Cell 3' v3.1 Gel Beads | 10x Genomics | 2000164 |
| Chromium Next GEM Chip G | 10x Genomics | 2000177 |
| Dual Index Plate TT Set A | 10x Genomics | 3000431 |
| Library Construction Kit | 10x Genomics | 1000190 |
| KAPA Library Quant Kit | Roche/KAPA | KK4873 |
| SPRIselect Reagent | Beckman Coulter | B23318 |
| High Sensitivity D5000 Tapestation Cassette | Agilent Technologies | 5067-5592 |
| High Sensitivity D5000 Tapestation Buffer | Agilent Technologies | 5190-7745 |
| High Sensitivity D5000 Tapestation Ladder | Agilent Technologies | 5190-7747 |
| KAPA SYBR FAST qPCR Master Mix | Illumina | KK4600 |
| | | |
| IHC antibodies | | |
| CD8α XP Rabbit mAB | Cell Signaling Technology | 98941 |
| | | |
| Chemicals, peptides, and recombinant proteins | | |
| DNase I | Roche/Sigma | 10104159001 |
| Dispase | Gibco | 17105-041 |
| Collagenase P | Roche/Sigma | 11249002001 |
| Tris-HCl (pH 7.4) | Sigma Aldrich | T2194 |
| Sodium Chloride (NaCl) | Sigma Aldrich | S5150 |
| Magnesium Chloride (MgCl2) | Sigma Aldrich | M1028 |
| 10% BSA in DPBS | Sigma Aldrich | A1595-50ML |
| Low TE Buffer | Thermo Fisher | 12090-015 |
| Tris 1M pH 7.0 | Millipore Sigma | T1819-100ML |
| 10% Tween 20 | Bio-Rad | 1662404 |
| Buffer EB | Qiagen | 1014608 |
| ViaStain AOPI Staining Solution | Nexcelom | CS2-0106-5mL |
